# Epilepsy-associated *SCN2A* (Na_V_1.2) variants exhibit diverse and complex functional properties

**DOI:** 10.1085/jgp.202313375

**Published:** 2023-08-14

**Authors:** Christopher H. Thompson, Franck Potet, Tatiana V. Abramova, Jean-Marc DeKeyser, Nora F. Ghabra, Carlos G. Vanoye, John J. Millichap, Alfred L. George

**Affiliations:** 1 Department of Pharmacology, Feinberg School of Medicine, Northwestern University, Chicago, IL, USA; 2 Department of Neurology, Feinberg School of Medicine, Northwestern University, Chicago, IL, USA

## Abstract

Pathogenic variants in voltage-gated sodium (Na_V_) channel genes including *SCN2A*, encoding Na_V_1.2, are discovered frequently in neurodevelopmental disorders with or without epilepsy. *SCN2A* is also a high-confidence risk gene for autism spectrum disorder (ASD) and nonsyndromic intellectual disability (ID). Previous work to determine the functional consequences of *SCN2A* variants yielded a paradigm in which predominantly gain-of-function variants cause neonatal-onset epilepsy, whereas loss-of-function variants are associated with ASD and ID. However, this framework was derived from a limited number of studies conducted under heterogeneous experimental conditions, whereas most disease-associated *SCN2A* variants have not been functionally annotated. We determined the functional properties of *SCN2A* variants using automated patch-clamp recording to demonstrate the validity of this method and to examine whether a binary classification of variant dysfunction is evident in a larger cohort studied under uniform conditions. We studied 28 disease-associated variants and 4 common variants using two alternatively spliced isoforms of Na_V_1.2 expressed in HEK293T cells. Automated patch-clamp recording provided a valid high throughput method to ascertain detailed functional properties of Na_V_1.2 variants with concordant findings for variants that were previously studied using manual patch clamp. Many epilepsy-associated variants in our study exhibited complex patterns of gain- and loss-of-functions that are difficult to classify by a simple binary scheme. The higher throughput achievable with automated patch clamp enables study of variants with greater standardization of recording conditions, freedom from operator bias, and enhanced experimental rigor. This approach offers an enhanced ability to discern relationships between channel dysfunction and neurodevelopmental disorders.

## Teaser

Hundreds of genetic variants in the voltage-gated sodium channel Na_V_1.2 are associated with a spectrum of neurodevelopmental disorders with or without epilepsy. High throughput automated patch-clamp recording of several variants revealed more complex patterns of dysfunction than previously recognized.

## Introduction

Epilepsy and neurodevelopmental disorders (NDD) with clinical onset during infancy and childhood are often attributed to monogenic etiologies (Lindy et al., 2018; Symonds et al., 2019; Symonds and McTague, 2020). Among the dozens of individual genes associated with these conditions are several that encode voltage-gated ion channels including sodium and potassium channels expressed throughout the nervous system. Defining the functional consequences of ion channel variants in this disease context can inform a mechanistic framework that advances understanding of pathophysiology and helps guide conceptualization of targeted therapeutic approaches (Brunklaus et al., 2022).

*SCN2A* encodes a voltage-gated sodium channel (Na_V_1.2) expressed widely in the developing and mature brain. Pathogenic *SCN2A* variants are associated with childhood-onset epilepsy of varying severity as well as autism spectrum disorder (ASD) with or without accompanying seizures and nonsyndromic intellectual disability (ID; Sanders et al., 2018; Hedrich et al., 2019; Wolff et al., 2019; Meisler et al., 2021). The type of variant in these conditions differs to some extent, with truncating variants (premature stop codons, frameshifts) being most prevalent in ASD/ID whereas missense variants being more common in persons with epilepsy. Because truncating *SCN2A* variants likely produce nonfunctional Na_V_1.2 channels, and because earlier studies of missense variants associated with epilepsy demonstrated gain-of-function (GoF), a general genotype–phenotype correlation emerged (Sanders et al., 2018). Specifically, evidence from in vitro functional evaluation of *SCN2A* variants supported the hypothesis that loss-of-function (LoF) variants were the drivers of ASD/ID, whereas GoF variants caused early-onset epilepsy (Ben-Shalom et al., 2017). This genotype–phenotype correlation for *SCN2A* provided a framework to predict which individuals might respond best to sodium channel–blocking anti-seizure medications. Seizures may also occur in individuals with *SCN2A* LoF variants, and evidence from mouse models suggests that maladaptive changes in potassium channel expression in the brain may offer an explanation (Spratt et al., 2021; Zhang et al., 2021).

Distinguishing GoF and LoF based on in vitro studies of recombinant Na_V_1.2 channels can be challenging because of the myriad of functional properties that can be assessed. This is particularly the case with missense variants of uncertain significance. Variants that fail to generate measurable sodium current are easy to categorize as LoF. Variants that affect the time course or voltage dependence of channel gating may also be simple to characterize as GoF in some cases. For example, a depolarizing shift in the voltage dependence of inactivation or a hyperpolarizing shift in the voltage dependence of activation will promote a greater open probability of sodium channels. Abnormal inactivation (e.g., slower time course, enhanced persistent current) is also recognized as a GoF property exhibited by some variant sodium channels. When these dysfunctional properties occur in isolation, it is straightforward to assign GoF or LoF to a variant. However, variants with complex mixtures of gain and loss of individual functional properties are more difficult to categorize.

In this study, we determined the functional properties of several *SCN2A* variants mostly associated with neonatal onset epilepsy using automated patch-clamp recording, which enables higher throughput than traditional manual patch clamp. We validated this approach by studying known benign and pathogenic variants, then applied the technique to a set of variants that were not previously studied. We observed a range of functional properties among the variants with a substantial fraction exhibiting complex or mixed patterns of dysfunction. Our findings suggest a need to re-evaluate the simple binary classification of *SCN2A* variants and to engage in efforts to determine how variants with mixed properties promote neuronal dysfunction.

## Materials and methods

### Cell culture

HEK293T cells (CRL-3216; American Type Culture Collection) were stably transfected with the human sodium channel β_1_ (SCN1B) and β_2_ (SCN2B) auxiliary subunits facilitated by *piggyBac* transposon-mediated genome insertion (Kahlig et al., 2010) The resulting cell line (HEK-β cells) was maintained in Dulbecco’s modified Eagle’s medium (GIBCO/Invitrogen) supplemented with 10% fetal bovine serum (Atlanta Biologicals), 2 mM *L*-glutamine, 50 U/ml penicillin, and 50 μg/ml streptomycin at 37°C in 5% CO_2_.

### Plasmids and mutagenesis

Full-length cDNA encoding WT or variant intron-stabilized human Na_V_1.2 corresponding to both adult (NCBI accession NM_021007) and neonatal (NCBI accession NM_001371246) splice isoforms (Thompson et al., 2020; DeKeyser et al., 2021) was engineered into vectors in which we introduced a high-efficiency encephalomyocarditis virus internal ribosome entry site (IRES) with A6 bifurcation sequence (Bochkov and Palmenberg, 2006) followed by the monomeric red fluorescent protein mScarlet (pIRES2-mScarlet).

Variants were introduced into Na_V_1.2 by site-directed mutagenesis using Q5 2X high-fidelity DNA polymerase Master Mix (New England Biolabs) as previously described (DeKeyser et al., 2021). Mutagenic primers were designed using custom software (available upon request) and are presented in Table S1.

### Electroporation

For automated electrophysiology experiments, plasmids encoding WT or variant Na_V_1.2 were electroporated into HEK-β cells using the MaxCyte STX system (MaxCyte, Inc.; Vanoye et al., 2021). Cells were grown to 70–80% confluence and harvested using TrypLE (Thermo Fisher Scientific). A 500 μl aliquot of cell suspension was used to determine cell number and viability using an automated cell counter (ViCell, Beckman Coulter). The remaining cells were collected by gentle centrifugation (193 × *g*, 4 min) at room temperature, followed by washing the cell pellet with 5 ml electroporation buffer (EBR100; MaxCyte, Inc.). Cells were resuspended at a final density of 10^8^ viable cells/ml.

For electroporations, 50 μg of WT or variant Na_V_1.2 was mixed with 100 μl of cell suspension (10^8^ cells/ml). The DNA–cell suspension mix was then transferred to an OC-100 × 2 processing assembly (MaxCyte, Inc.) and electroporated using the Optimization 4 protocol. Immediately after electroporation, 10 μl DNase I (Sigma-Aldrich) was added to the DNA–cell suspension mix and the entire mixture was transferred to a 60-mm dish and incubated for 30 min at 37°C in 5% CO_2_. Following incubation, cells were gently resuspended and grown in a T75 flask for 48 h at 37°C in 5% CO_2_. Cells were then harvested, transfection efficiency was determined by flow cytometry (see below), and then frozen in 1 ml aliquots at 1.8 × 10^6^ viable cells/ml. For manual patch-clamp recording experiments, HEK-β cells were transiently transfected with WT or variant Na_V_1.2 (2 μg) using Qiagen SuperFect reagent (Qiagen).

### Flow cytometry

Transfection efficiency following electroporation was assessed prior to cell freezing using a BD FACSCanto II flow cytometer (BD Biosciences). Forward scatter (FSC), side scatter (SSC), and red fluorescence (phyocoerythrin channel, PE) were recorded. A 561-nm excitation laser was used. FSC and SSC were used to gate single viable cells and eliminate doublets, dead cells, and debris. 10,000 events were recorded for each sample, and non-transfected HEK-β cells were used as a negative control. Data were analyzed using BD FACSDiva 8.0.2.

### Cell preparation for automated electrophysiology

Electroporated cells were thawed 1 d before experiments and grown ∼24 h at 37°C in 5% CO_2_. Prior to experiments, cells were dispersed using TrypLE and a 500 μl aliquot was taken to determine cell number and viability by automated cell counting. Cells were gently centrifuged (193 × *g*) for 4 min at room temperature and resuspended at a final density of 180,000 viable cells/ml in an external recording solution (see below). Cells were allowed to recover on a shaking rotating platform (200 rpm) at 15°C for 15–30 min prior to recording.

### Automated electrophysiology

**Figure S1.**
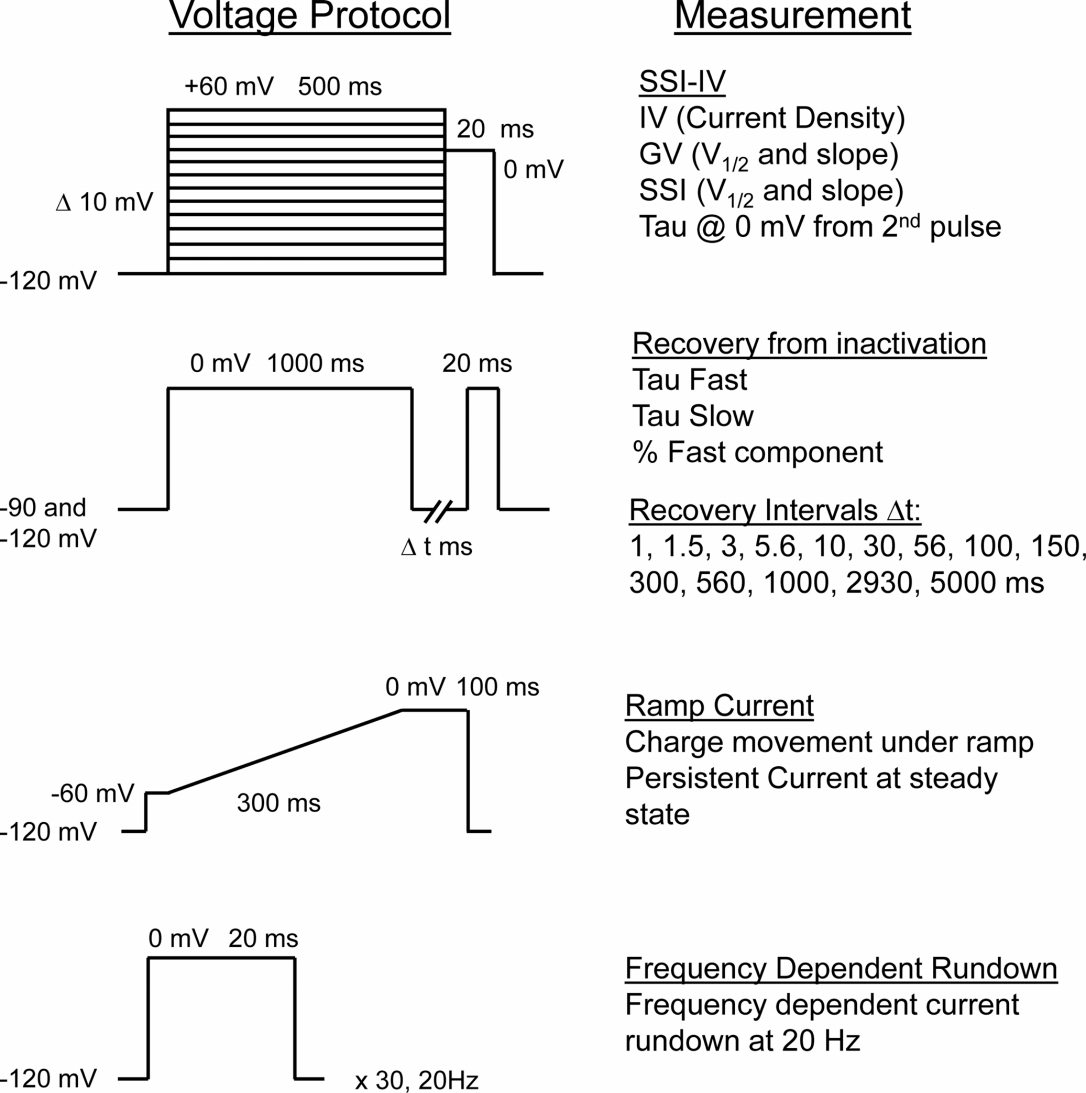
Voltage protocols used to assess voltage-gated sodium channel biophysical parameters by both automated and manual electrophysiology.

Automated patch-clamp recording was performed using the Nanion SyncroPatch 768PE platform (Nanion Technologies; Vanoye et al., 2021). Single-hole low resistance (2–3.5 MΩ) recording chips were used for this study. Pulse generation and data collection were performed using PatchControl384 v1.6.6 and DataControl384 v1.6.0 software (Nanion Technologies). Whole-cell currents were acquired at 10 kHz, series resistance was compensated 80%, and leak currents were subtracted using P/4 subtraction. Liquid junction potential was corrected at the beginning of each experiment. Whole-cell currents were recorded at room temperature using voltage protocols illustrated in Fig. S1. The external solution contained (in mM) 140 NaCl, 4 KCl, 2 CaCl_2_, 1 MgCl_2_, 10 HEPES, and 5 glucose, with the final pH adjusted to 7.4 with NaOH and osmolality adjusted to 300 mOsm/kg with sucrose. The composition of the internal solution was (in mM) 110 CsF, 10 CsCl, 10 NaCl, 20 EGTA, and 10 HEPES, with the final pH adjusted to 7.2 with CsOH and osmolality adjusted to 300 mOsm/kg with sucrose. High-resistance seals were obtained by addition of 10 μl seal enhancer solution comprised of (in mM) 125 NaCl, 3.75 KCl, 10.25 CaCl_2_, 3.25 MgCl_2_, and 10 HEPES, with final pH adjusted to 7.4 with NaOH, followed immediately by addition of 30 μl of external solution to each well. Prior to recording, cells were washed twice with external solution and the final concentrations of CaCl_2_ and MgCl_2_ were 3 and 1.3 mM, respectively.

Stringent criteria were used to select cells for inclusion in the final analysis (seal resistance ≥200 MΩ, access resistance ≤20 MΩ, capacitance ≥2 pF, and sodium reversal potential between 45 and 85 mV). The average reversal potential for Na_V_1.2 expressing cells in our final dataset is 70.3 ± 0.3 mV, which is close to the predicted reversal potential of 68.2 based on the composition of intracellular and extracellular solutions. All biophysical data were collected from cells whose currents were larger than −200 pA. Ramp currents were analyzed only when recorded from cells having peak currents larger than −2 nA and seal resistances ≥1 GΩ. Voltage control was assessed from conductance–voltage (GV) curves and cells were included in the final analysis if two adjacent points on the GV curve showed no more than a sevenfold increase. Cells with corrected voltage error >5 mV were excluded. Unless otherwise noted, all chemicals were obtained from Sigma-Aldrich.

A typical experiment was recorded from cells expressing WT-Na_V_1.2 and either five Na_V_1.2 variants or four Na_V_1.2 variants plus nontransfected cells seeded into 64-well clusters (four 16-well columns) of a 384-well patch clamp plate. Cells recorded at the same time were electroporated with either WT or variant plasmids in parallel on the same day. To ensure that sufficient numbers of cells were recorded to account for attrition from stringent quality control data filters, we recorded from two 384-well plates simultaneously. Because both plates were run simultaneously, and the plate layout was identical, we combined data from both plates and normalized the data for each variant to the average WT values recorded on the same day. Biophysical properties were listed as not determined (ND) if less than five replicates were obtained for that property for any given variant.

### Manual patch-clamp recording

Whole-cell voltage-clamp experiments of heterologous cells were performed as previously described (Thompson et al., 2020). Recordings were made at room temperature using an Axopatch 200B amplifier (Molecular Devices). Liquid junction potential was corrected at the beginning of each experiment, series resistance was compensated by 90%, and leak currents were subtracted using a P/4 protocol. Cells with corrected voltage error >2.5 mV were excluded. Patch pipettes were pulled from borosilicate glass capillaries (Harvard Apparatus Ltd.) with a multistage P-1000 Flaming-Brown micropipette puller (Sutter Instruments Co.) and fire-polished using a microforge (MF-830; Narashige) to a resistance of 1.5–2.5 MΩ. The pipette solution consisted of (in mM) 110 CsF, 10 CsCl, 10 NaCl, 20 EGTA, and 10 HEPES, with the final pH adjusted to 7.2 with CsOH and osmolality adjusted to 300 mOsm/kg with sucrose. Cells in the recording chamber were superperfused with bath solution containing (in mM) 140 NaCl, 4 KCl, 3 CaCl_2_, 2 MgCl_2_, 1 HEPES, and 5 glucose, with the final pH adjusted to 7.4 with NaOH and osmolality adjusted to 310 mOsm/kg with sucrose.

### Data analysis

Data were analyzed and plotted using a combination of DataControl384 v1.6.0 (Nanion Technologies), Clampfit 10.4 (Molecular Devices), Microsoft Excel (Microsoft Office 2019), and GraphPad Prism (GraphPad Software). Averaged normalized traces for display purposes were created using the following steps. First, we averaged raw current traces (not corrected for cell capacitance) for WT and variants from a single experiment. Then we normalized the averaged traces to the maximal inward current for the WT averaged trace. For quantitative analysis, whole-cell currents were normalized to membrane capacitance. GraphPad Prism was used to fit voltage dependence of activation and inactivation curves with Boltzmann functions, and recovery from inactivation with a two-exponential equation. Window current was calculated by integrating the area under the intersection between the Boltzmann fits for voltage dependence of activation and inactivation using a custom MatLab script (Ganguly et al., 2021). All data were normalized to WT run in parallel and data were treated as not normally distributed for statistical analyses. Unless otherwise noted, data are presented as mean ± 95% confidence intervals (CI). Statistical comparison of manual and automated recording of Na_V_1.2A-WT was performed using a Mann–Whitney test. Statistical analyses of Na_V_1.2 variants were performed using a Kruskal–Wallis test followed by Dunn’s post-hoc test for multiple comparisons. The threshold for statistical significance was P ≤ 0.05. Exact P values are presented in the supplemental tables.

### Online supplemental material

**Figure S2.**
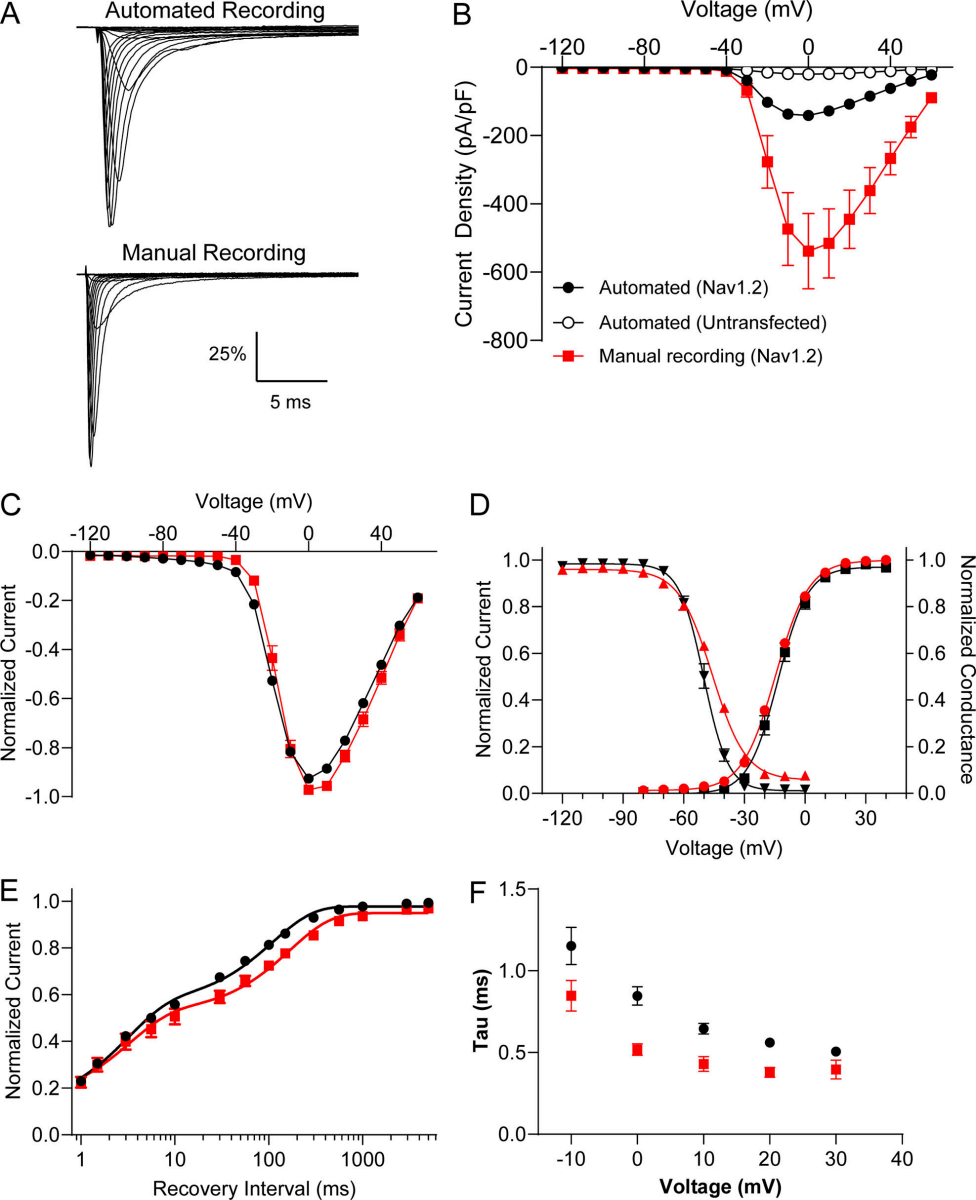
**Comparison of automated and manual patch-clamp evaluation of Na**_**V**_**1.2. (A)** Average normalized whole-cell sodium currents from automated (top) and manual (bottom) patch-clamp recording of cells expressing WT Na_V_1.2A. **(B)** Summary of current–voltage relationships for automated recording of untransfected cells (open circles), automated recording of cell expressing Na_V_1.2A (black circles), and manual recording of cells expressing Na_V_1.2A (red squares). **(C)** Normalized whole-cell sodium currents comparing automated (black circles) and manual (red squares) recording methods. **(D)** Voltage-dependence of activation and inactivation of Na_V_1.2A comparing automated (black) and manual (red) recording methods. **(E)** Recovery from inactivation of Na_V_1.2A comparing automated (black circles) and manual (red squares) recording methods. **(F)** Time constant for the onset of inactivation of Na_V_1.2A as a function of membrane potential for automated (black circles) and manual (red squares) recording methods. All data are expressed as mean ± SEM from 14 to 1,253 cells. Error bars for automated patch recording data are obscured by symbols.

**Figure S3.**
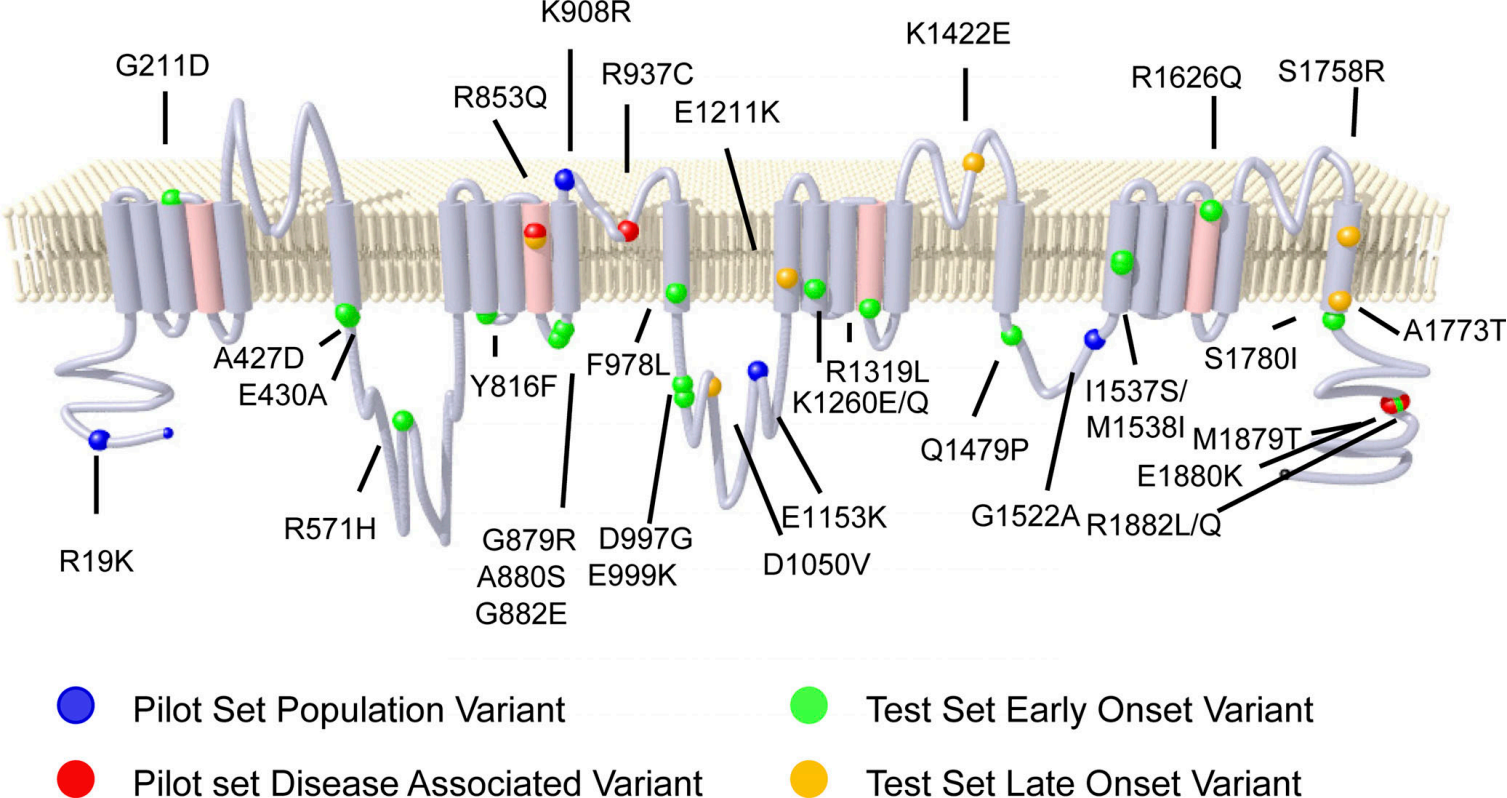
**Location of Na**_**V**_**1.2 variants.** Topology diagram of Na_V_1.2 showing population (blue spheres), known pathogenic (red spheres), early-onset (green spheres), and late-onset (orange spheres) epilepsy associated variants.

**Figure S4.**
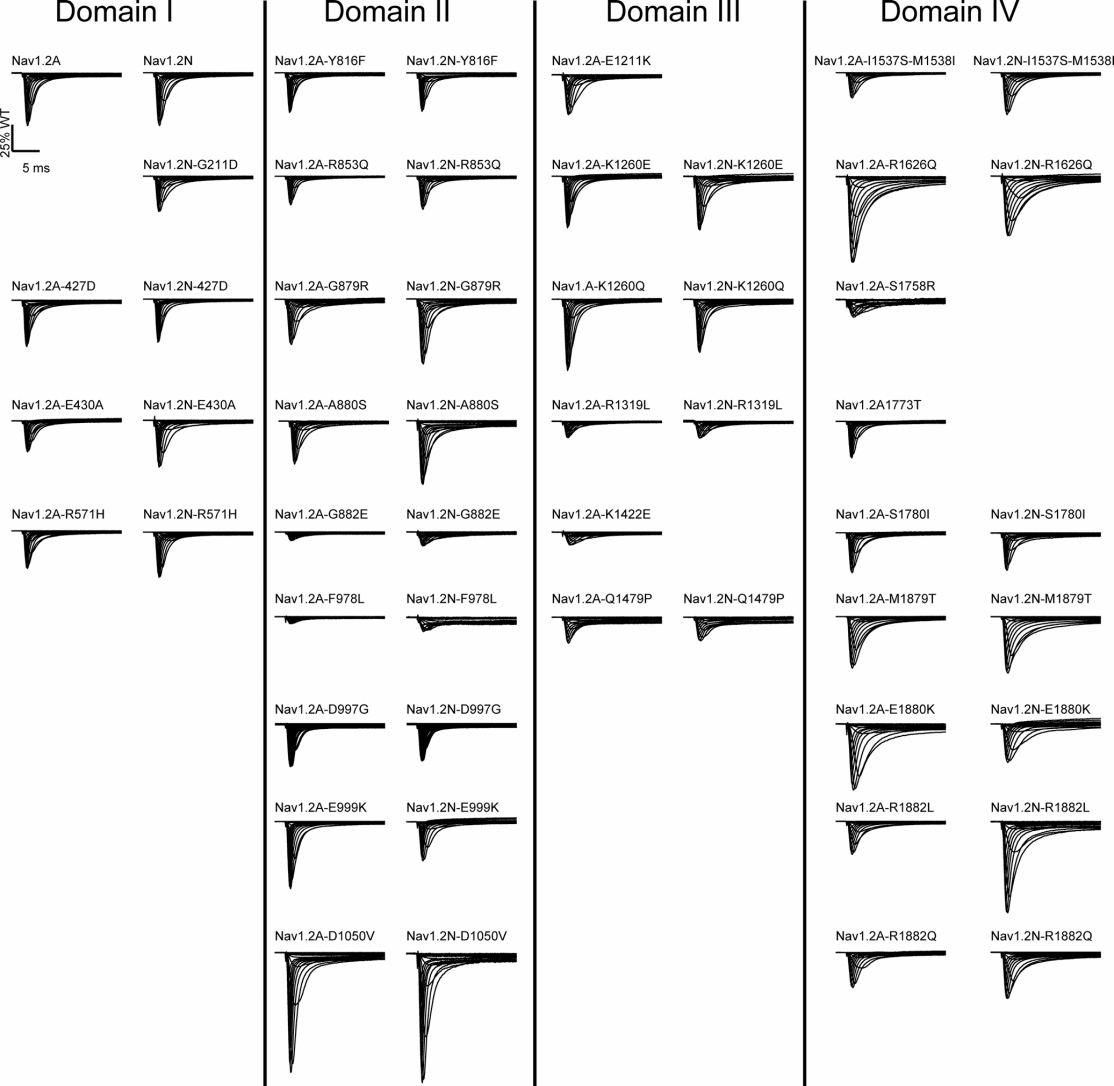
**Average normalized whole-cell currents of Na**_**V**_**1.2 variants.** Averaged whole-cell sodium currents (not corrected for cell capacitance) of Na_V_1.2 variants organized by domain. Currents from the adult and neonatal splice isoforms of each variant are shown side-by-side. All average traces were from 5 to 77 cells.

**Figure S5.**
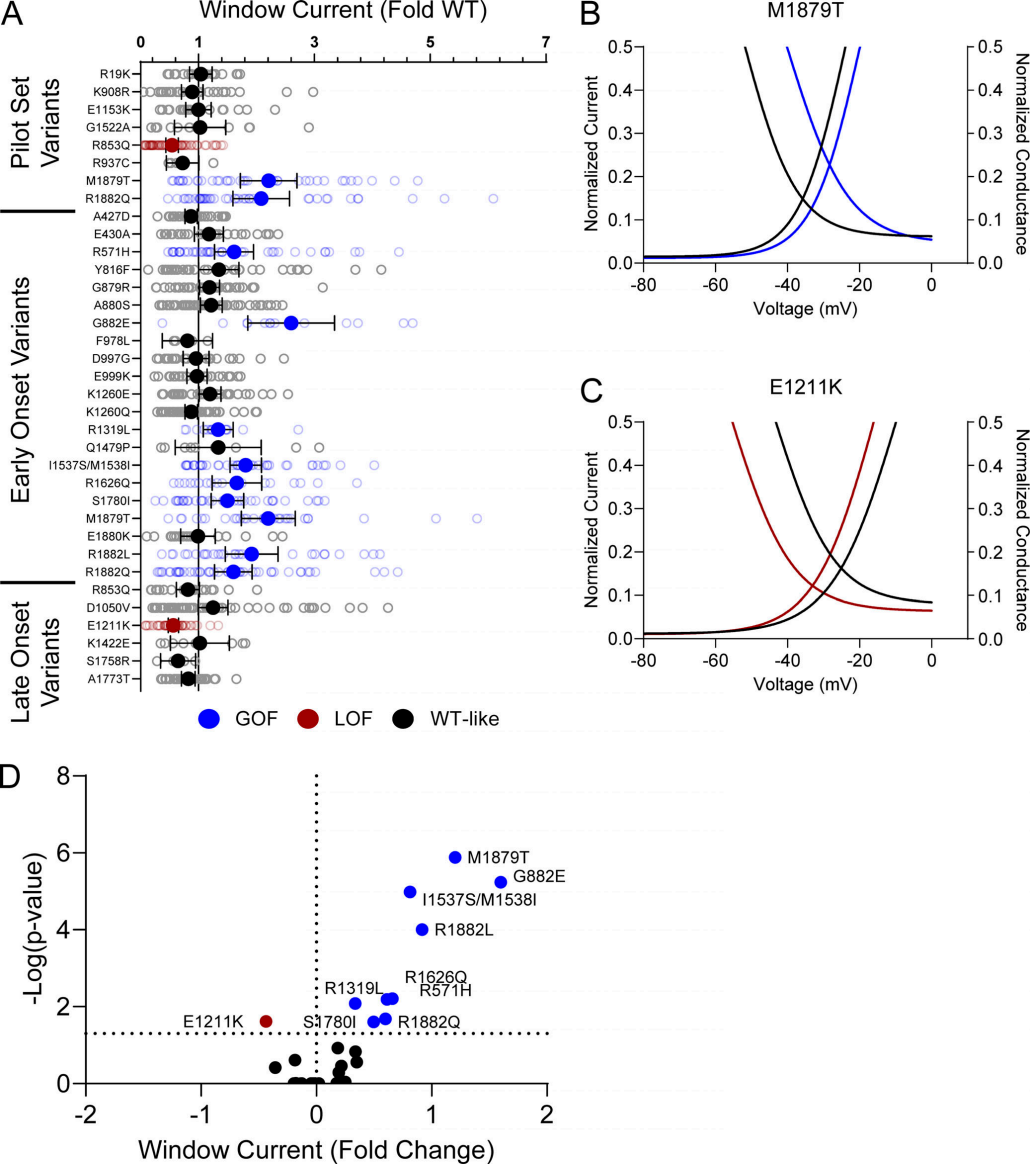
**Na**_**V**_**1.2 variants affect window current. (A)** Average deviation from WT Na_V_1.2A for the window current area. Boltzmann fit lines of representative variants showing. Data are plotted as mean ± 95% CI. **(B and C)** GoF; M1879T (B) or LoF; E1211K (C) window current respective to WT. **(D)** Volcano plot highlighting variants significantly different from WT. Red symbols and lines denote LoF and blue symbols and lines denote GoF with P < 0.05 (*n* = 5–57). Data were collected from two to four separate 384-well automated patch clamp experiments, and statistical comparisons were performed using a Kruskal–Wallis test followed by Dunn’s post-hoc test for multiple comparisons. Exact P values are presented in Tables S3 and S4.

**Figure S6.**
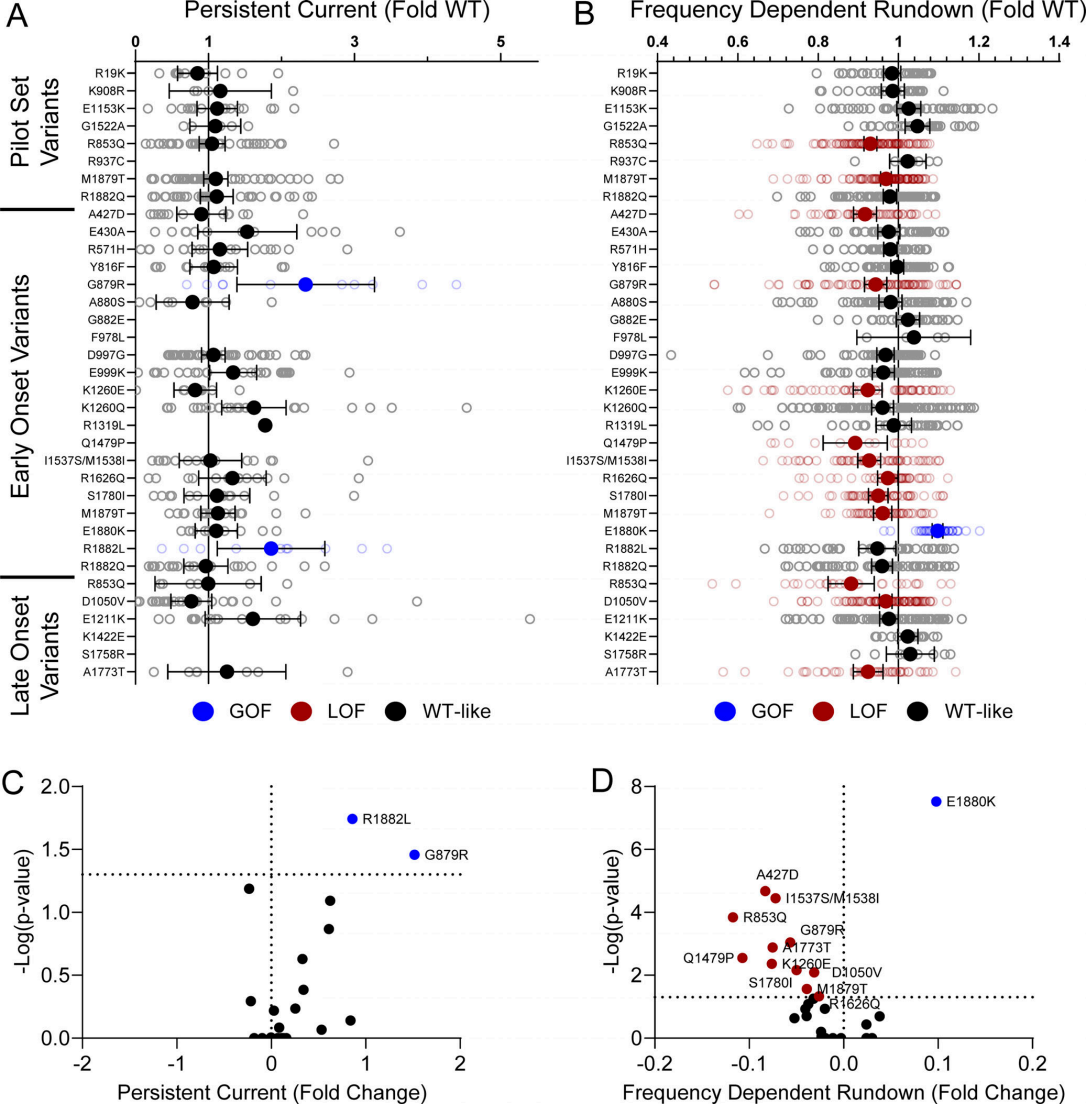
**Na**_**V**_**1.2 variants affect channel inactivation. (A and B)** Average deviation from WT Na_V_1.2A for (A) persistent current and (B) frequency-dependent channel rundown at 20 Hz. Data are plotted as mean ± 95% CI. **(C and D)** Volcano plots highlighting variants significantly different from WT for (C) persistent current and (D) frequency-dependent rundown. Red symbols denote LoF and blue symbols denote GoF with P < 0.05 (*n* = 5–103). Data were collected from two to four separate 384-well automated patch clamp experiments, and statistical comparisons were performed using a Kruskal–Wallis test followed by Dunn’s post-hoc test for multiple comparisons. Exact P values are presented in Tables S3 and S4.

**Figure S7.**
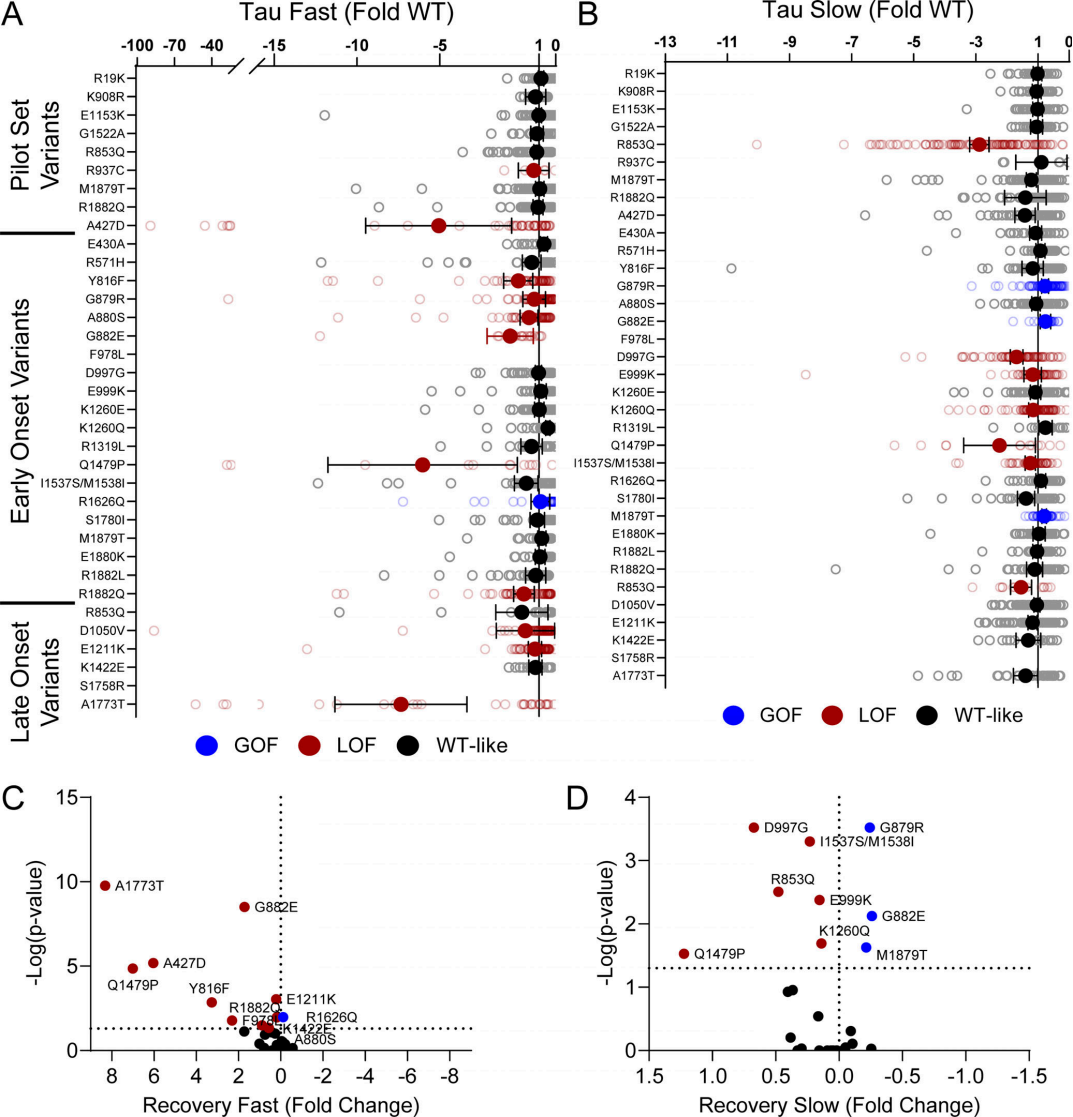
**Na**_**V**_**1.2 variants affect recovery from inactivation. (A and B)** Average deviation from WT Na_V_1.2A for the (A) fast and (B) slow time constants of recovery from inactivation. **(C and D)** Volcano plots highlighting variants significantly different from WT for (C) fast and (D) slow time constants of recovery from inactivation. Red symbols denote LoF and blue symbols denote GoF with P < 0.05 (*n* = 13–97). Data were collected from two to four separate 384-well automated patch clamp experiments, and statistical comparisons were performed using a Kruskal–Wallis test followed by Dunn’s post-hoc test for multiple comparisons. Exact P values are presented in Tables S3 and S4.

**Figure S8.**
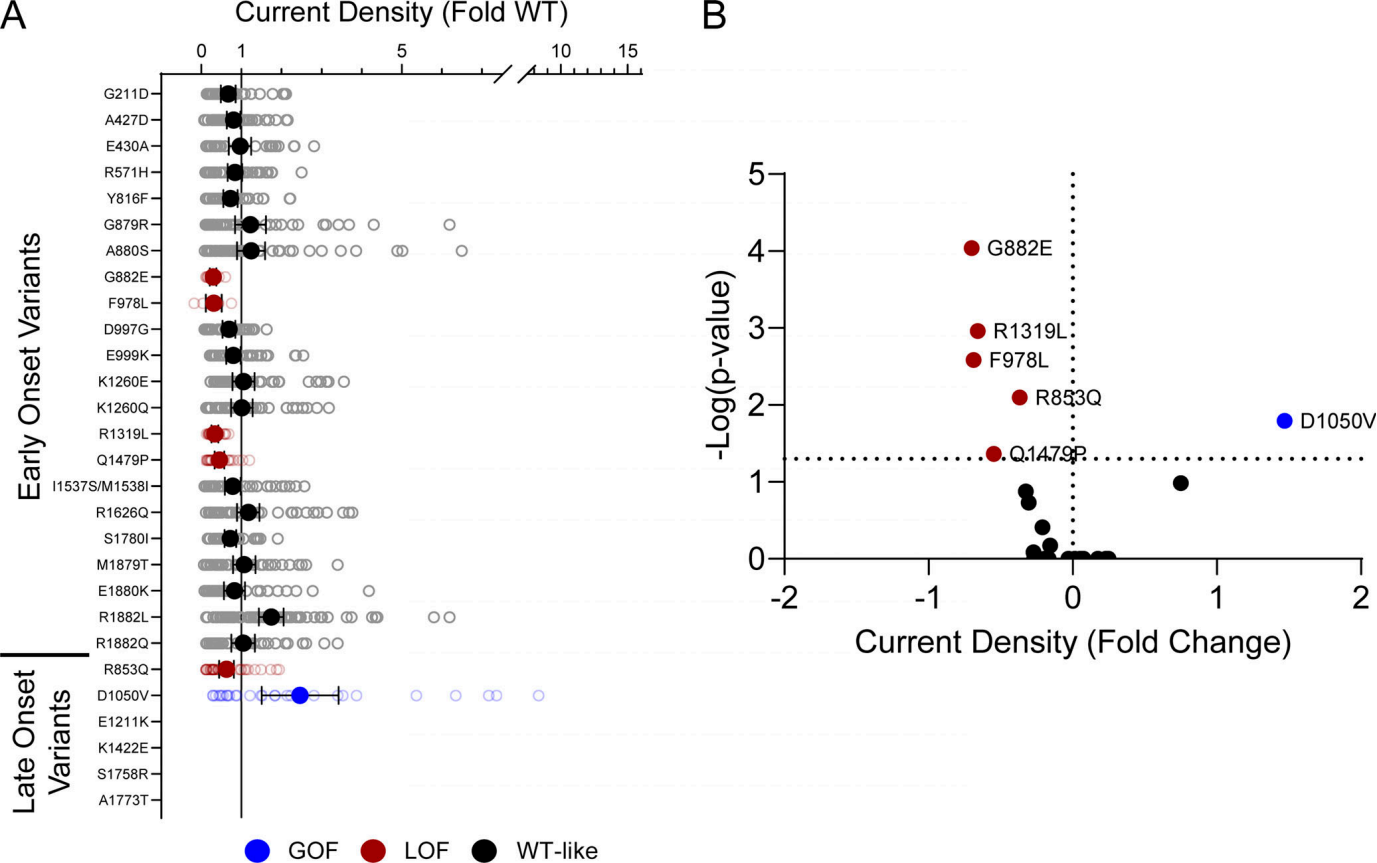
**Disease-associated variants affect neonatal Na**_**V**_**1.2 whole-cell currents. (A)** Average deviation of whole-cell sodium current density from neonatal WT Na_V_1.2N for epilepsy-associated variants. Data are plotted as mean ± 95% CI. **(B)** Volcano plot highlighting variants significantly different from WT. Red symbols denote LoF and blue symbols denote GoF with P < 0.05 (*n* = 9–76). Data were collected from two to four separate 384-well automated patch clamp experiments, and statistical comparisons were performed using a Kruskal–Wallis test followed by Dunn’s post-hoc test for multiple comparisons. Exact P values are presented in Table S5.

**Figure S9.**
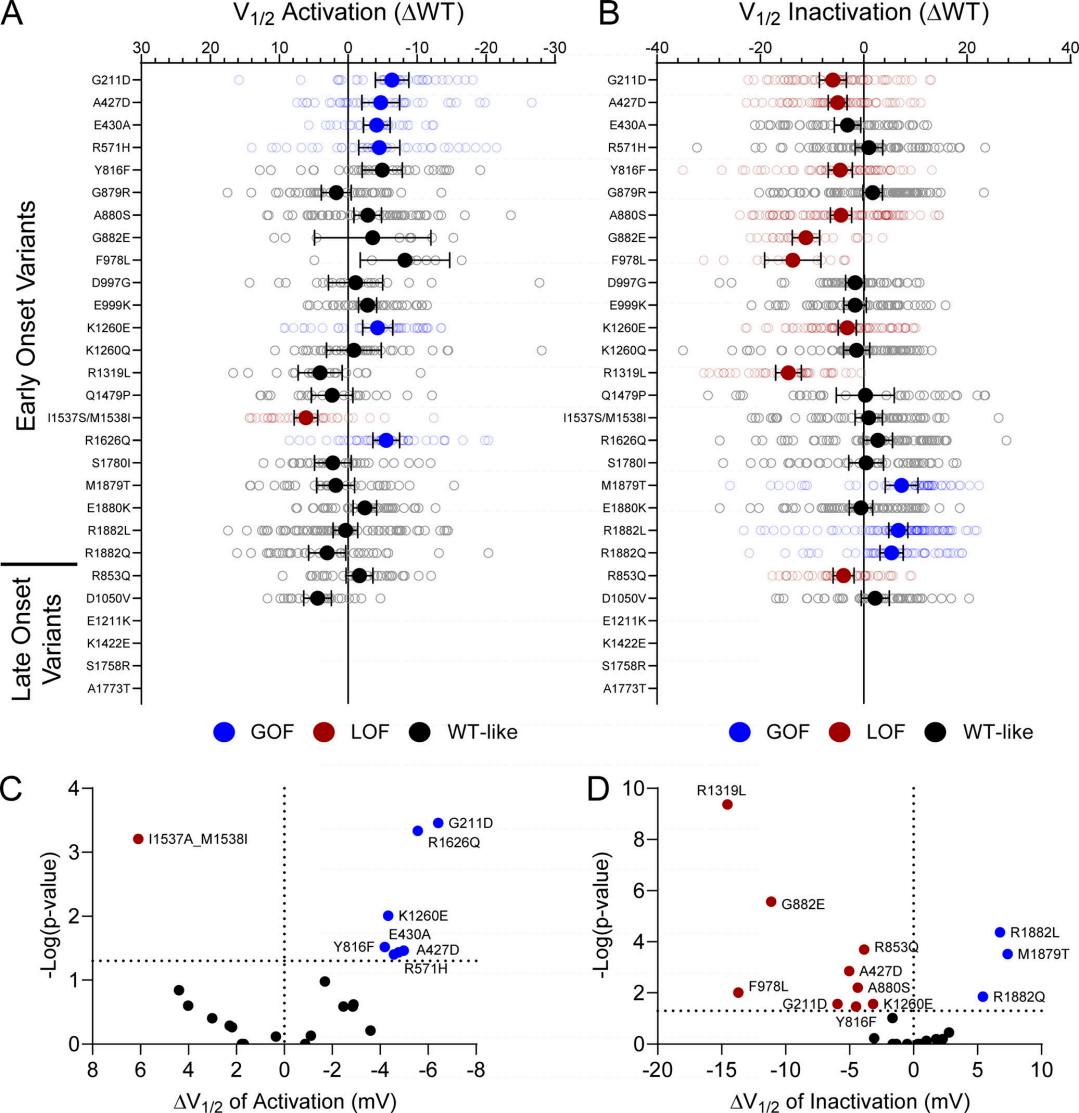
**Disease-associated variants affect neonatal Na**_**V**_**1.2 voltage-dependence of activation and inactivation. (A and B)** Average deviation from neonatal WT Na_V_1.2N for V_1/2_ of (A) activation and (B) steady-state inactivation (in mV). Data are plotted as mean ± 95% CI. **(C and D)** Volcano plots highlighting variants significantly different from WT for voltage dependence of (C) activation and (D) inactivation. Red symbols denote LoF and blue symbols denote GoF with P < 0.05 (*n* = 7–95). Data were collected from two to four separate 384-well automated patch clamp experiments, and statistical comparisons were performed using a Kruskal–Wallis test followed by Dunn’s post-hoc test for multiple comparisons. Exact P values are presented in Table S5.

**Figure S10.**
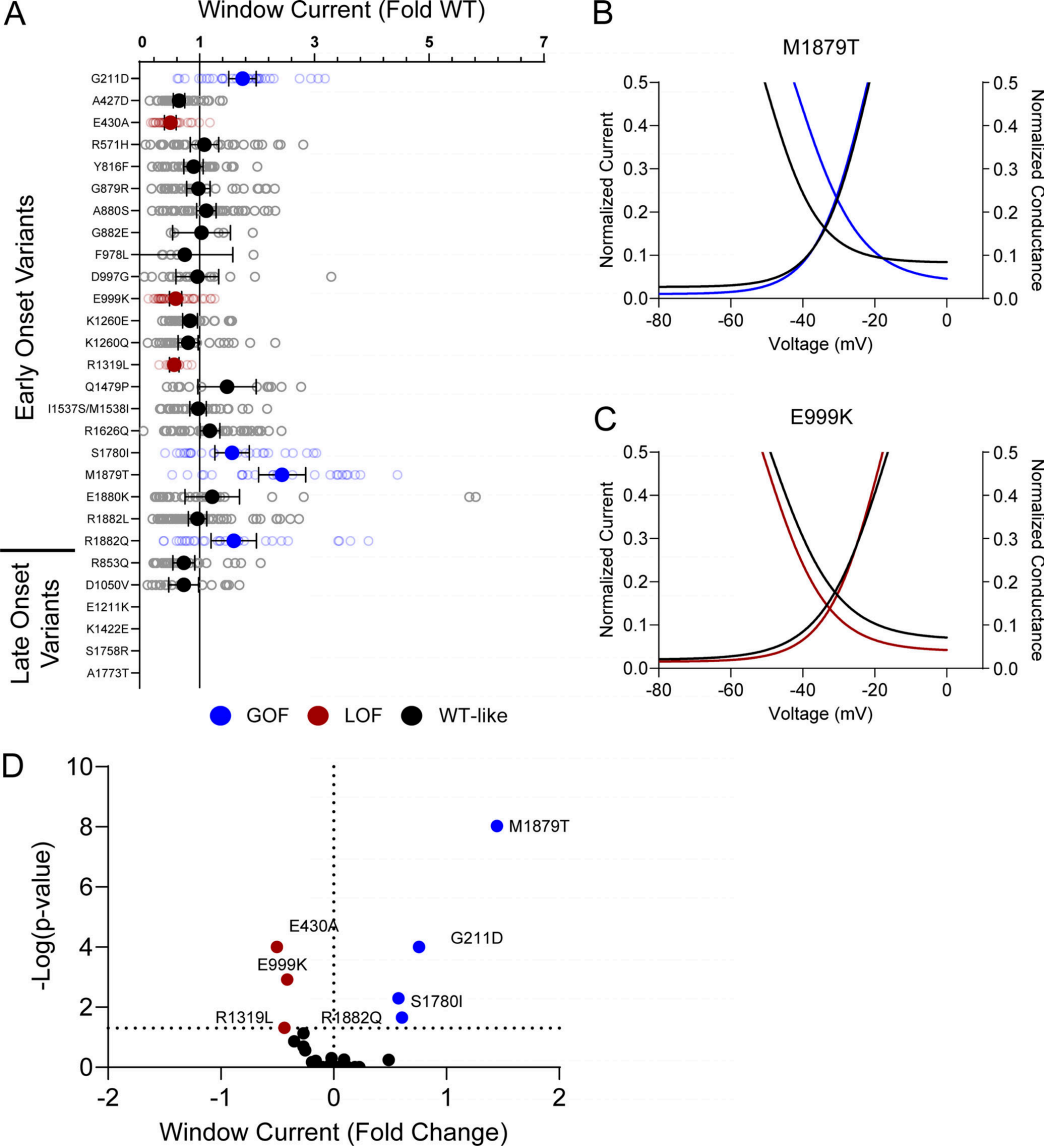
**Disease-associated variants affect neonatal Na**_**V**_**1.2 affect window current. (A)** Average deviation from WT Na_V_1.2N for the window current area. Data are plotted as mean ± 95% CI. **(B and C)** Boltzmann fit lines of representative variants showing (B) GoF; M1879T or (C) LoF; E999K window current respective to WT. **(D)** Volcano plot highlighting variants significantly different from WT. Red symbols and lines denote LoF and blue symbols and lines denote GoF with P < 0.05 (*n* = 5–59). Data were collected from two to four separate 384-well automated patch clamp experiments, and statistical comparisons were performed using a Kruskal–Wallis test followed by Dunn’s post-hoc test for multiple comparisons. Exact P values are presented in Table S5.

**Figure S11.**
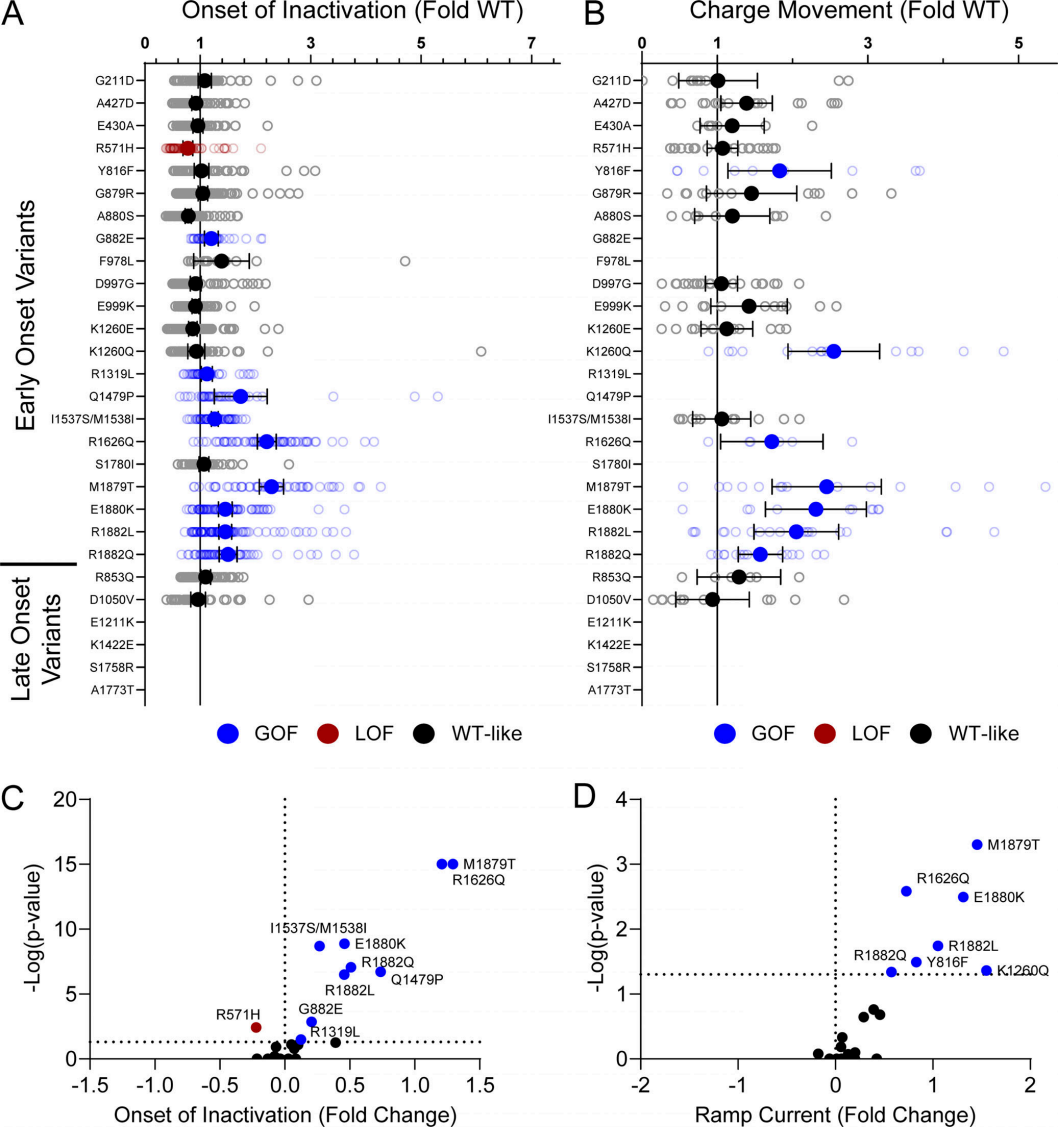
**Disease-associated variants affect neonatal Na**_**V**_**1.2 affect inactivation time constants and ramp currents. (A and B)** Average deviation of (A) inactivation time constant (τ) and (B) ramp currents from neonatal WT Na_V_1.2N for epilepsy associated variants. Data are plotted as mean ± 95% CI. Red symbols denote LoF and blue symbols denote GoF with P < 0.05 (*n* = 6–101). Data were collected from two to four separate 384-well automated patch clamp experiments, and statistical comparisons were performed using a Kruskal–Wallis test followed by Dunn’s post-hoc test for multiple comparisons. Exact P values are presented in Table S5.

**Figure S12.**
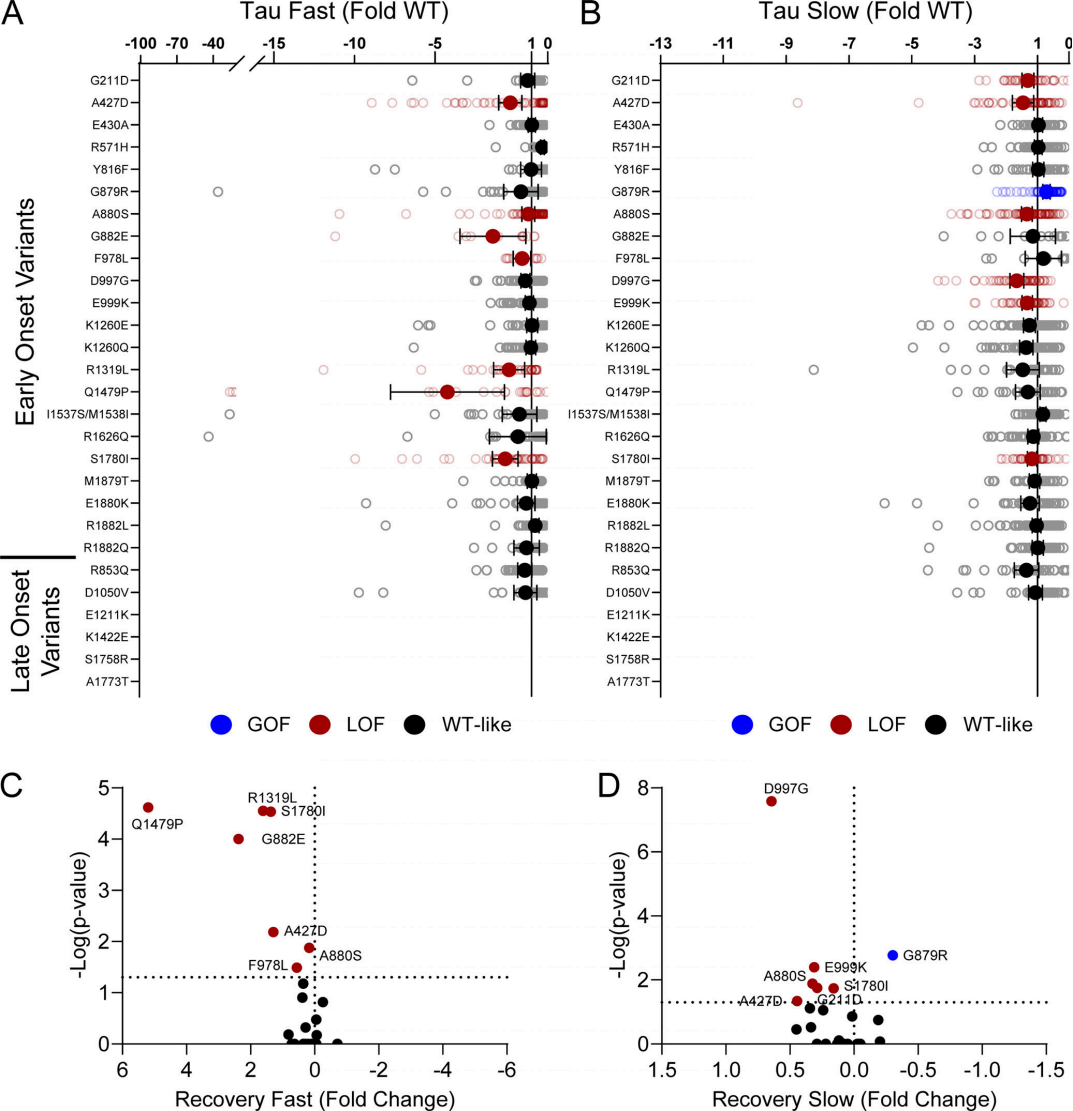
**Disease-associated variants affect neonatal Na**_**V**_**1.2 affect recovery from inactivation. (A and B)** Average deviation from neonatal WT Na_V_1.2N for the (A) fast and (B) slow time constants of recovery from inactivation. Data are plotted as mean ± 95% CI. **(C and D)** Volcano plots highlighting variants significantly different from WT for (C) fast and (D) slow time constants of recovery from inactivation. Red symbols denote LoF and blue symbols denote GoF with P < 0.05 (*n* = 12–84). Data were collected from two to four separate 384-well automated patch clamp experiments, and statistical comparisons were performed using a Kruskal–Wallis test followed by Dunn’s post-hoc test for multiple comparisons. Exact P values are presented in Table S5.

**Figure S13.**
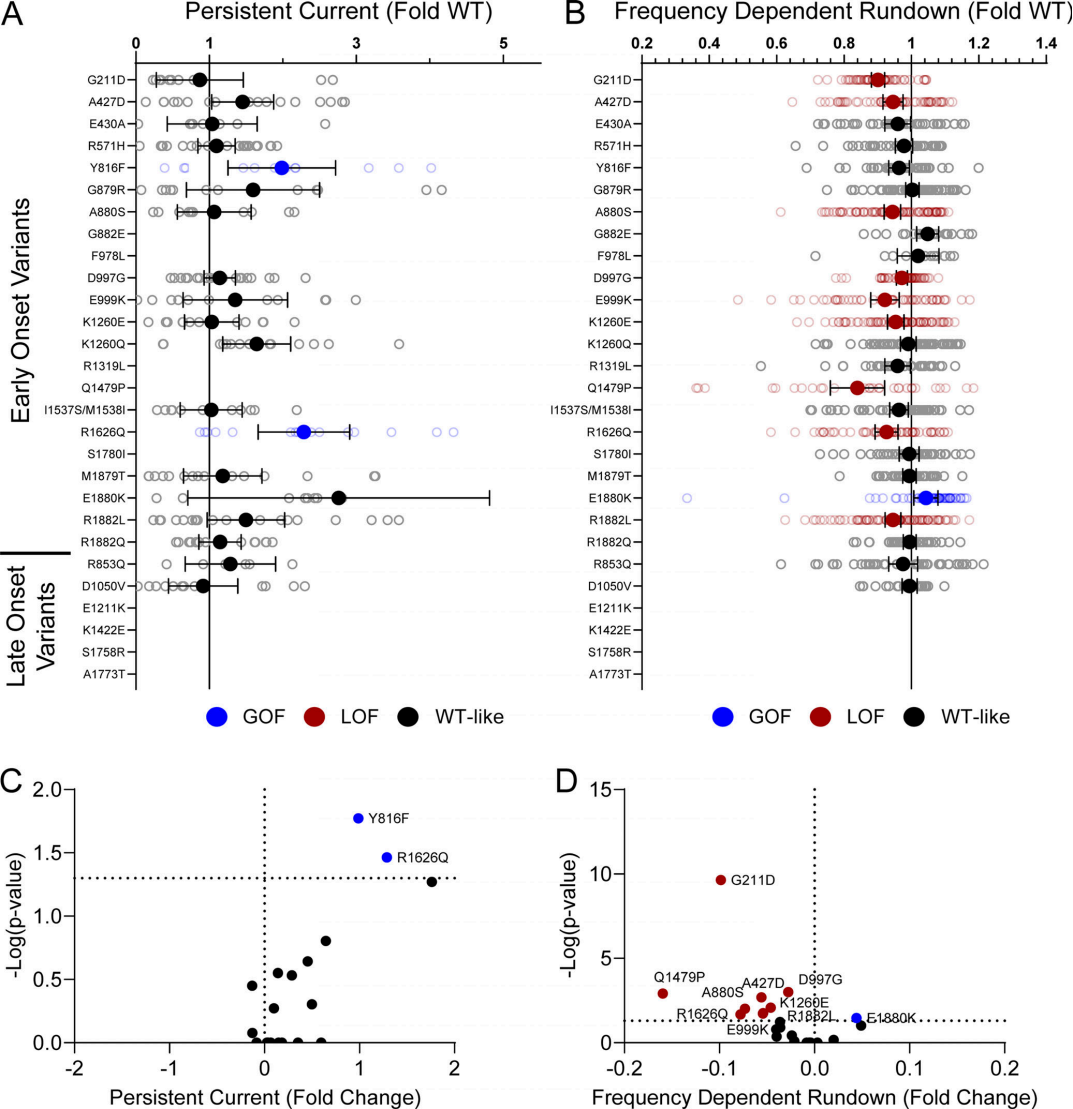
**Disease-associated variants affect neonatal Na**_**V**_**1.2 affect persistent current and frequency-dependent channel rundown. (A and B)** Average deviation from neonatal WT Na_V_1.2N for (A) persistent sodium current and (B) frequency-dependent channel rundown at 20 Hz. Data are plotted as mean ± 95% CI. **(C and D)** Volcano plots highlighting variants significantly different from WT for (C) persistent current and (D) frequency-dependent rundown. Red symbols denote LoF and blue symbols denote GoF with P < 0.05 (*n* = 6–88). Data were collected from two to four separate 384-well automated patch clamp experiments, and statistical comparisons were performed using a Kruskal–Wallis test followed by Dunn’s post-hoc test for multiple comparisons. Exact P values are presented in Table S5.

**Figure S14.**
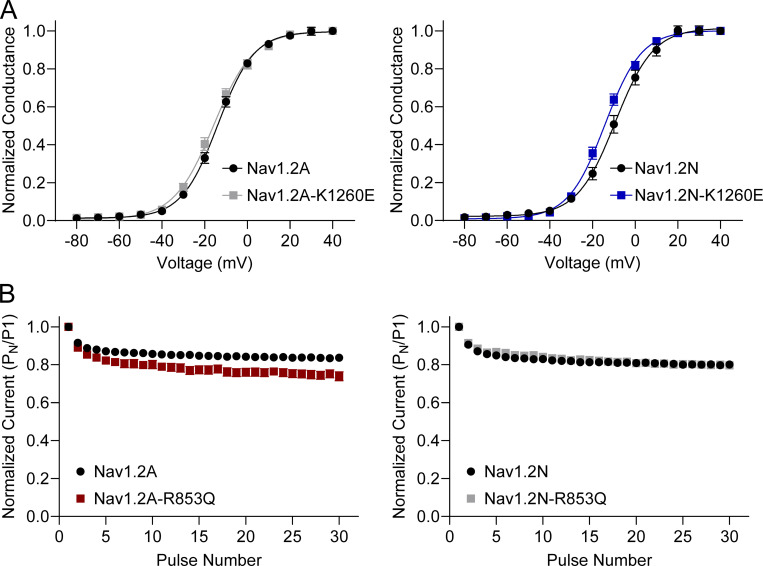
**Na**_**V**_**1.2 variants exhibit splice isoform-dependent functional properties. (A)** Voltage dependence of activation of adult (left) and neonatal (right) splice isoforms of K1260E. **(B)** Frequency-dependent channel rundown of adult (left) and neonatal (right) splice isoforms of R853Q. All data are expressed as mean ± SEM of 27–88 cells.

Fig. S1 shows the voltage protocols used to assess voltage-gated sodium channel biophysical parameters. Fig. S2 gives the comparison of automated and manual patch-clamp evaluation of Na_V_1.2. Fig. S3 shows the location of Na_V_1.2 variants. Fig. S4 provides the average normalized whole-cell currents of Na_V_1.2 variants. Fig. S5 demonstrates that Na_V_1.2 variants affect window current. Fig. S6 demonstrates that Na_V_1.2 variants affect channel inactivation. Fig. S7 demonstrates that Na_V_1.2 variants affect recovery from inactivation. Fig. S8 demonstrates that disease-associated variants affect neonatal Na_V_1.2 whole-cell currents. Fig. S9 demonstrates that disease-associated variants affect neonatal Na_V_1.2 voltage dependence of activation and inactivation. Fig. S10 demonstrates that disease-associated variants affect neonatal Na_V_1.2 affect window current. Fig. S11 demonstrates that disease-associated variants affect neonatal Na_V_1.2 affect inactivation time constants and ramp currents. Fig. S12 demonstrates that disease-associated variants affect neonatal Na_V_1.2 affect recovery from inactivation. Fig. S13 demonstrates that disease-associated variants affect neonatal Na_V_1.2 affect persistent current and frequency-dependent channel rundown. Fig. S14 demonstrates that Na_V_1.2 variants exhibit splice isoform-dependent functional properties. Table S1 is a list of mutagenesis primers used to generate the Na_V_1.2 variants. Tables S2, S3, S4, and S5 are complete sets of analyzed data presented in Excel format.

## Results

### Automated electrophysiological analysis of Na_V_1.2

We employed a high-efficiency electroporation method to transiently express recombinant human Na_V_1.2 suitable for automated electrophysiology. The Na_V_1.2 subunit was coupled to mScarlet expression and electroporation efficiency was quantified by flow cytometry. We constructed separate expression plasmids encoding either the canonical adult brain-expressed splice variant (Na_V_1.2A) or one with an alternative exon 5 predominantly expressed in the developing brain (neonatal Na_V_1.2; Na_V_1.2N). Transfection efficiencies of 60–80% were achieved in HEK293T cells stably transfected with β1 and β2 accessory subunits.

We recorded robust whole-cell sodium currents using a dual 384-well automated electrophysiology platform and compared results with experiments performed on the same cells using manual patch-clamp recording (Fig. S2, A and B). The average peak current density measured by automated patch clamp was smaller than that obtained by manual patch clamp (Fig. S2 B), but normalized current–voltage relationships were nearly identical between the two recording methods (Fig. S2 C). The voltage dependence of activation was not significantly different between recording methods (Fig. S2 D and Table S2), whereas other parameters showed small differences between recording methods (Fig. S2, D–F; and Table S2). To control for nonspecific factors such as cell passage number, we designed automated patch clamp experiments to compare Na_V_1.2 variants only to the WT channel electroporated and assayed simultaneously. Electrophysiological data from 5,858 cells were analyzed for this study.

### Analytical validity of automated patch clamp for Na_V_1.2

**Figure 1.**
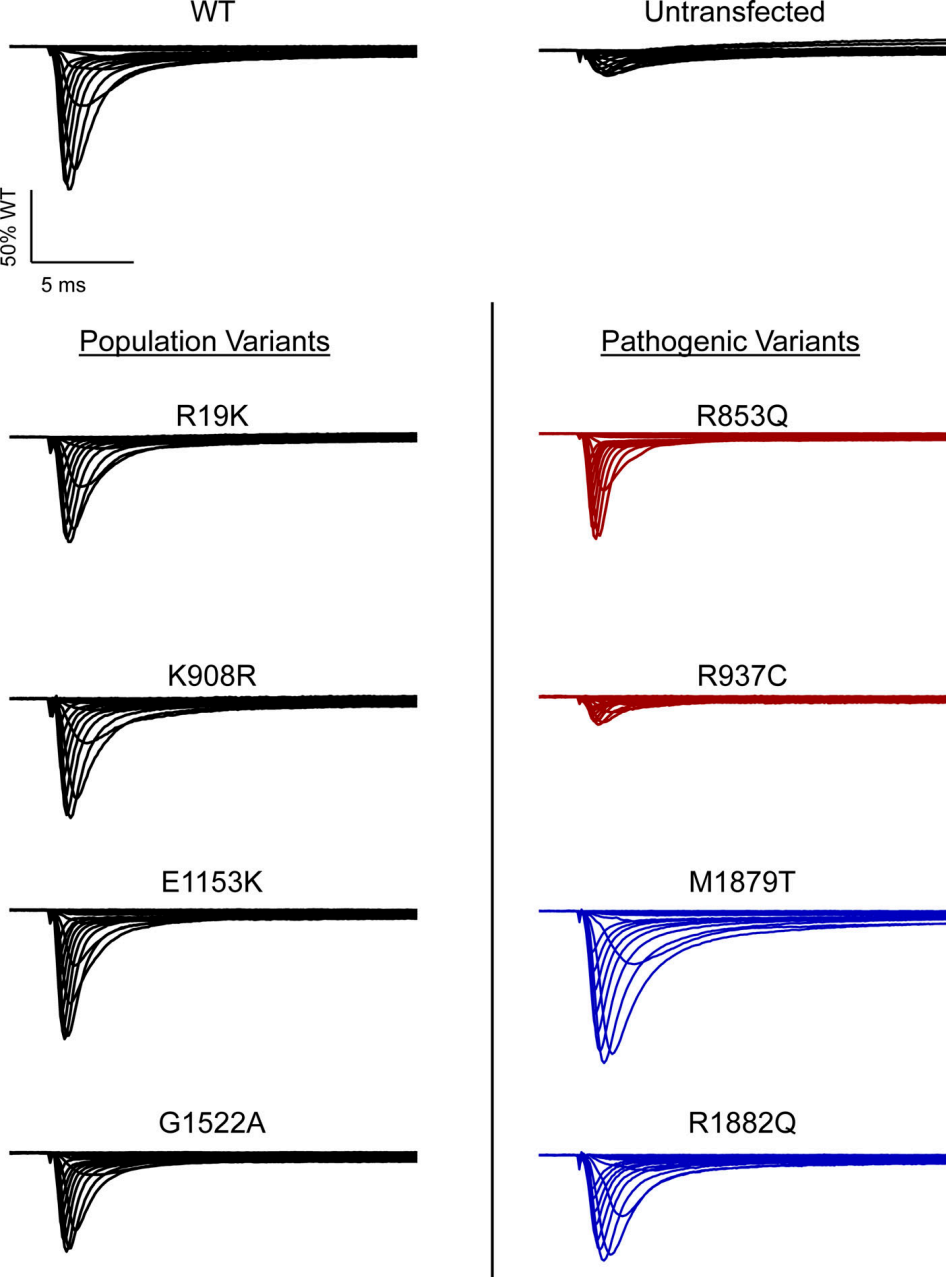
**Functional validation of a training set of Na**_**V**_**1.2 variants. (A–C)** Averaged whole-cell sodium current traces (not corrected for cell capacitance) of (A) WT Na_V_1.2A (left) untransfected cells (right), (B) a set of population variants, and (C) known pathogenic variants representing LOF (red) and GOF (blue) phenotypes. Traces were normalized to the average peak WT current amplitude. Average traces are from 5 to 65 cells.

To demonstrate analytical validity of automated patch clamp for Na_V_1.2, we engineered a collection of nonsynonymous population variants (R19K, K908R, E1153K, and G1522A) and previously published pathogenic missense variants (R853Q, R937C, M1879T, and R1882Q) in Na_V_1.2A to serve as a validation set (Fig. 1 and Fig. S3). Population variants were chosen based on minor allele frequency >0.0001 in the gnomAD database (Karczewski et al., 2020), while pathogenic variants were chosen to represent either GoF or LoF effects based on previously published data (Ben-Shalom et al., 2017; Berecki et al., 2018; Lauxmann et al., 2018; Begemann et al., 2019; Mason et al., 2019; Adney et al., 2020; Ganguly et al., 2021).

We measured several biophysical properties (Fig. S1) to fully capture the spectrum of sodium channel function, including whole-cell current density, voltage-dependence of activation and inactivation, recovery from inactivation, frequency-dependent channel rundown, time constant for the onset of inactivation at 0 mV, charge movement in response to a voltage ramp, and persistent sodium current. For all measured parameters, R19K, K908R, E1153K, and G1522A were indistinguishable from WT-Na_V_1.2A (Fig. 1 and Table S3).

Among the pathogenic variants chosen for the validation set, we chose two previously classified LoF variants (R853Q and R937C) and two GoF variants (M1879T and R1882Q). Our analysis of these variants using automated electrophysiology largely recapitulated previously published results with the exception of R853Q current density, which was not significantly different from WT channels (Table S3), whereas other properties conferring LoF were consistent with previous reports (Mason et al., 2019; Ganguly et al., 2021). For the other LoF variant, R937C, whole-cell sodium current (0.2 ± 0.01 of WT; Fig. 1 and Table S3) was indistinguishable from the current recorded from nontransfected cells (0.17 ± 0.01 of WT, Table S3). Automated patch-clamp recordings of the GoF variants M1879T and R1882Q in the validation set demonstrated similar patterns of dysfunction including depolarized shifts in the voltage-dependence of inactivation, slower inactivation time course, and a larger ramp current as reported previously (Fig. 1 and Table S3). Results from the validation set demonstrate that we can use an automated patch clamp to measure multiple GoF and LoF properties for pathogenic *SCN2A* variants and that nonpathogenic population variants are benchmarks for normal Na_V_1.2 function.

### Functional analysis of disease-associated Na_V_1.2 variants

We used this optimized approach to determine the functional consequences of 28 Na_V_1.2 variants identified through a patient registry established at The Ann and Robert H. Lurie Children’s Hospital of Chicago (Fig. S3; O’Connor et al., 2023 *Preprint*). This list included three of the previously mentioned pathogenic variants (R853Q, M1879T, and R1882Q) that are part of the validation set. Most of the variants (*n* = 22) were associated with developmental and epileptic encephalopathy (DEE) with seizure onset within the first month of life, whereas five were associated with DEE having seizure onset after 6 mo of age and one variant was associated with impaired neurodevelopment without seizures. One variant (G211D) affected a residue within the neonatal-expressed exon 5N and therefore was only studied in Na_V_1.2N. By contrast, E1211K, K1422S, S1758R, and A1773T were only studied in the canonical adult splice isoform because of the associated late clinical onset. All other variants were studied in both adult and neonatal Na_V_1.2 splice isoforms. We describe results for the variants expressed in Na_V_1.2A first.

**Figure 2.**
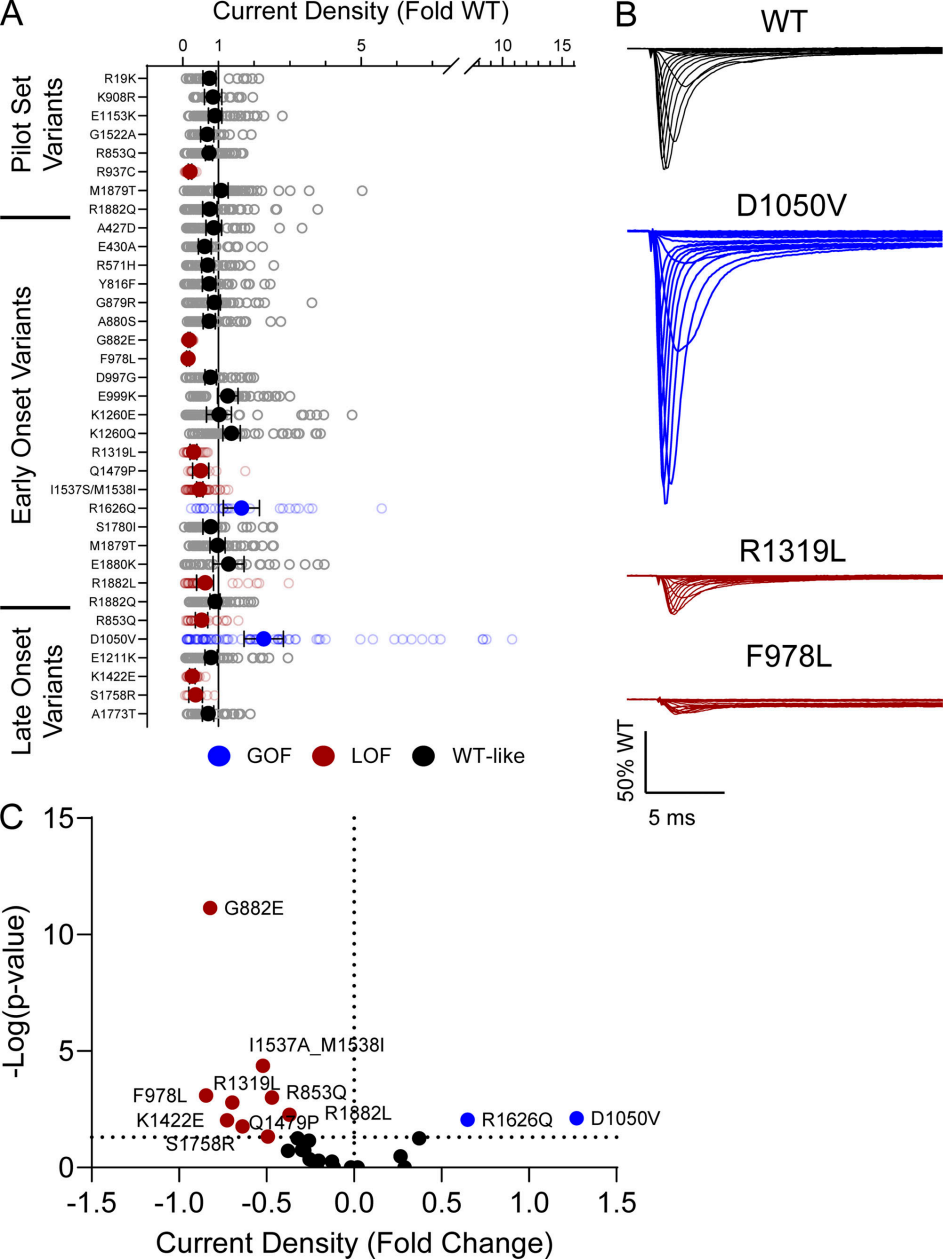
**Na**_**V**_**1.2 variants alter whole-cell current density. (A)** Average deviation of whole-cell sodium current density from WT Na_V_1.2A for population and disease-associated variants. Data are plotted as mean ± 95% CI. **(B)** Averaged whole-cell sodium current traces (not corrected for cell capacitance) for WT Na_V_1.2A, a GOF variant, D1050V, and two LOF variants, R1319L and F978L. Traces were normalized to the average peak WT current amplitude. **(C)** Volcano plot highlighting variants significantly different from WT. Red symbols denote variants classified as LoF and blue symbols denote variants classified as GoF based on current density significantly different from WT channels (*n* = 5–77). Data were collected from two to four separate 384-well automated patch clamp experiments, and statistical comparisons were performed using a Kruskal–Wallis test followed by Dunn’s post-hoc test for multiple comparisons. Exact P values are presented in Tables S3 and S4.

**Figure 3.**
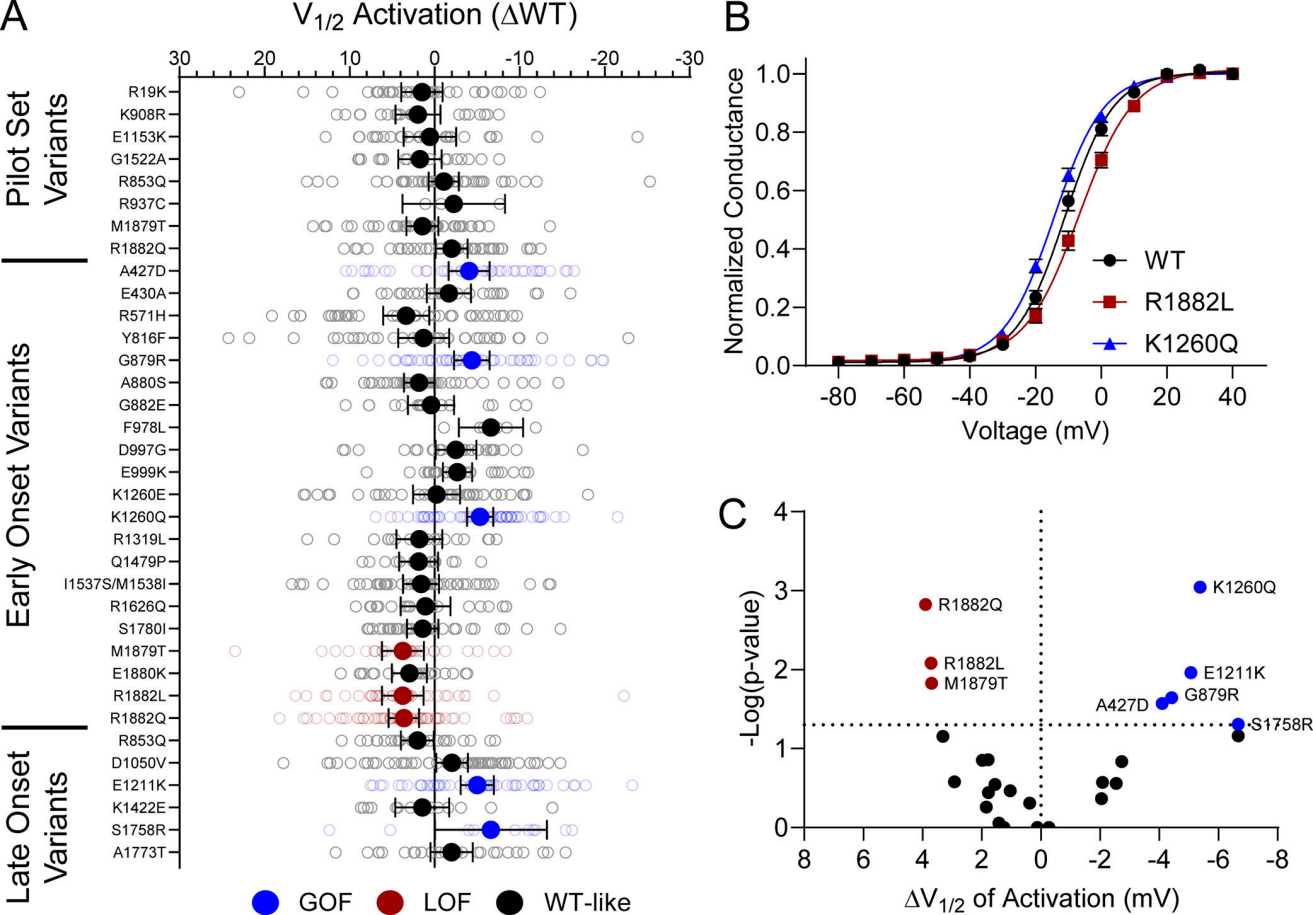
**Na**_**V**_**1.2 variants alter voltage-dependence of activation. (A)** Average deviation from WT Na_V_1.2A for V_½_ of activation (in mV). Data are plotted as mean ± 95% CI. **(B)** GV curves showing a GoF (K1260Q) and a LoF (R1882L) variant. Data are plotted as mean ± SEM. **(C)** Volcano plot highlighting variants significantly different from WT. Red symbols denote LoF and blue symbols denote GoF variants classified based on activation voltage dependence significantly different from WT channels (*n* = 5–58). Data were collected from two to four separate 384-well automated patch clamp experiments, and statistical comparisons were performed using a Kruskal–Wallis test followed by Dunn’s post-hoc test for multiple comparisons. Exact P values are presented in Tables S3 and S4.

**Figure 4.**
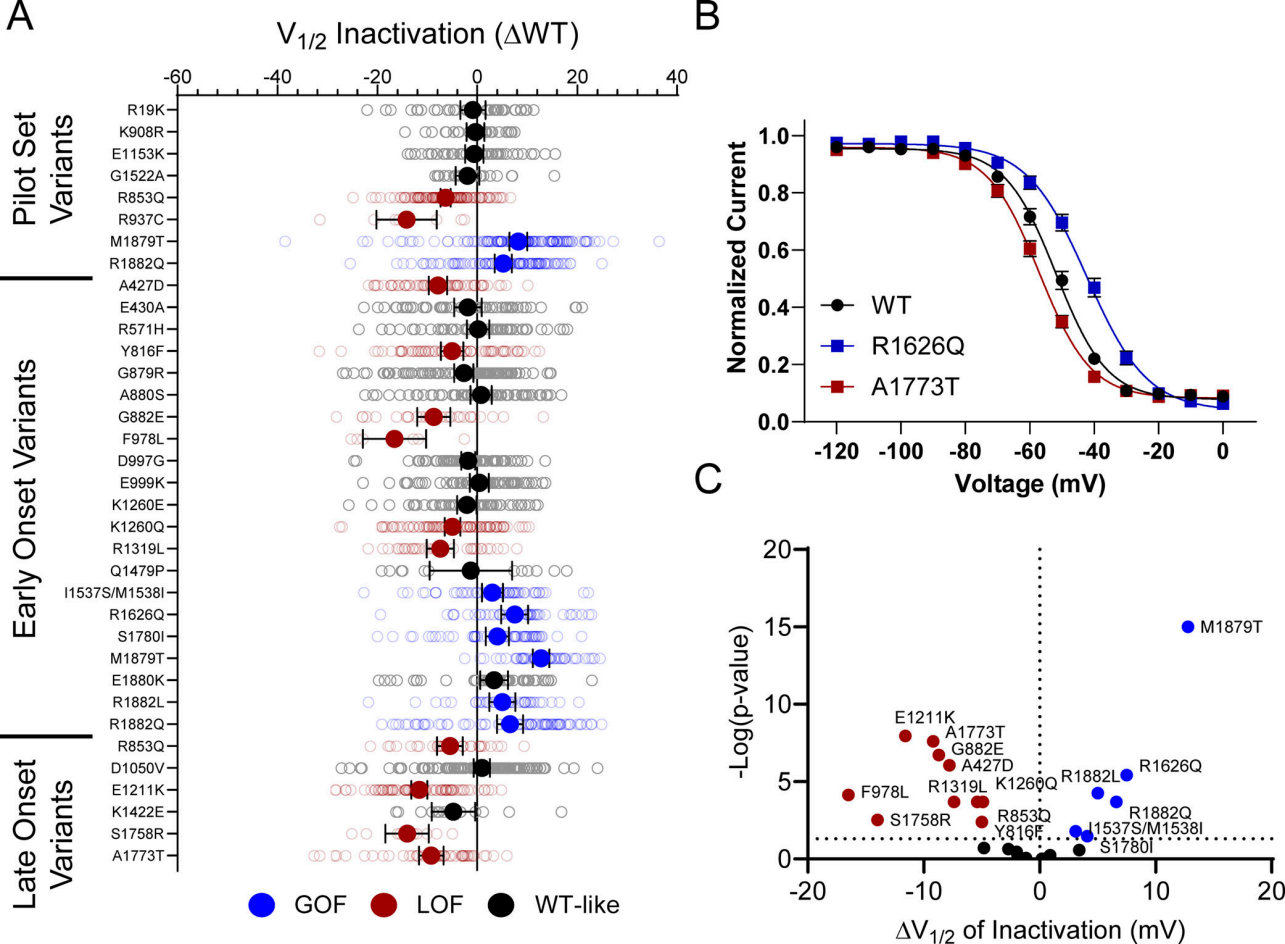
**Na**_**V**_**1.2 variants alter voltage dependence of inactivation. (A)** Average deviation from WT Na_V_1.2A for V_½_ of inactivation (in mV). Data are plotted as mean ± 95% CI. **(B)** Steady-state inactivation curves showing a GoF (R1626Q) and a LoF (A1773T) variant. Data are plotted as mean ± SEM. **(C)** Volcano plot highlighting variants significantly different from WT. Red symbols denote LoF and blue symbols denote GoF variants classified based upon inactivation voltage-dependent significantly different from WT channels (*n* = 8–122). Data were collected from two to four separate 384-well automated patch clamp experiments, and statistical comparisons were performed using a Kruskal–Wallis test followed by Dunn’s post-hoc test for multiple comparisons. Exact P values are presented in Tables S3 and S4.

10 of the analyzed variants expressed in the canonical Na_V_1.2A isoform exhibited significantly smaller peak current density than WT (Fig. 2, Fig. S4, and Table S4), ranging from a modest reduction (R1319L) to complete LoF (F978L; Fig. 2 B and Table S4). Two variants, D1050V and R1626Q, had current densities larger than WT (Fig. 2 B and Table S4). We observed significant differences in the voltage dependence of activation for eight variants with five exhibiting hyperpolarized shifts (A427D, G879R, E1211K, K1260Q, and S1758R), and three a depolarized shift (M1879T, R1882L, and R1882Q; Fig. 3 and Table S4). Voltage dependence of inactivation was significantly different from WT channels for 20 variants including 12 variants with hyperpolarized shifts (Fig. 4 and Table S4). Interestingly, there is a cluster of variants in domain IV and the C-terminus that all exhibited depolarized shifts in the voltage-dependence of inactivation (I1537S/M1538I, R1626Q, S1780I, M1879T, R1882L, and R1882Q; Fig. 4 and Table S4).

Voltage dependence of activation and inactivation curves overlap to define a region called the window current where channels are open and not inactivated. The presence of larger or smaller window currents may contribute to LoF or GoF driven by differences in voltage-dependent gating. Variants with larger depolarization of voltage dependence of inactivation, such as I1537S/M1538I, R1626Q, M1879T, R1882L, and R1882Q, exhibited larger window current compared with WT channels (Fig. S5, A and C; and Table S4). There were no variants with a hyperpolarized activation V_½_ that exhibited a larger window current. Interestingly, E1211K shows both a hyperpolarization of activation and inactivation. The resulting window current is smaller compared with WT and suggests that E1211K may result in an overall LoF with respect to voltage-dependent gating (Fig. S5, A and D; and Table S4).

**Figure 5.**
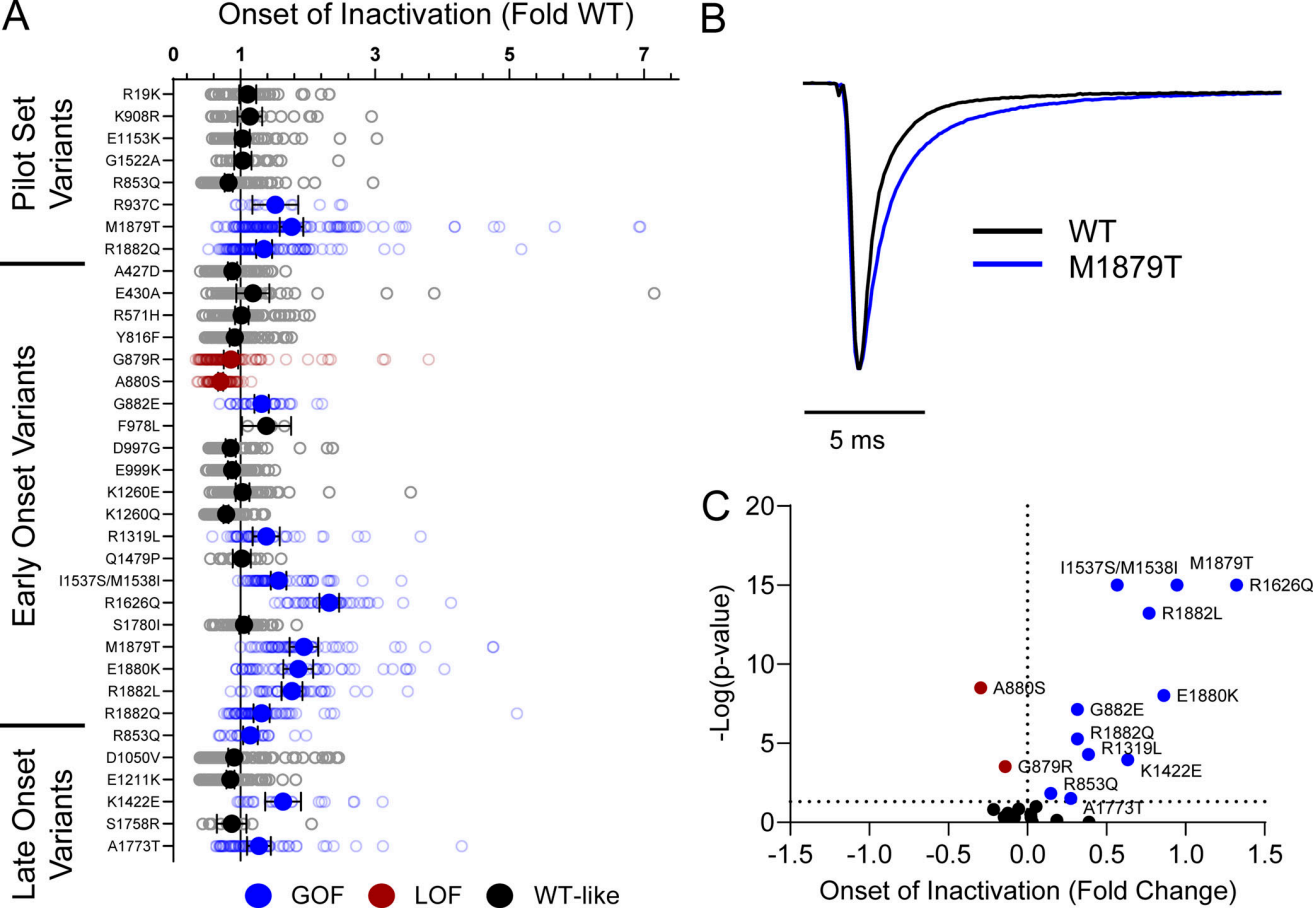
**Na**_**V**_**1.2 variants alter inactivation time constants. (A)** Average deviation of inactivation time constant (τ) measured at 0 mV from WT Na_V_1.2A for disease-associated variants. Data are plotted as mean ± 95% CI. **(B)** Average traces for WT Na_V_1.2A and a GoF variant M1879T recorded at 0 mV. **(C)** Volcano plot highlighting variants significantly different from WT. Red symbols denote LoF and blue symbols denote GoF variants classified based upon inactivation kinetics significantly different from WT channels (*n* = 9–120). Data were collected from two to four separate 384-well automated patch clamp experiments, and statistical comparisons were performed using a Kruskal–Wallis test followed by Dunn’s post-hoc test for multiple comparisons. Exact P values are presented in Tables S3 and S4.

**Figure 6.**
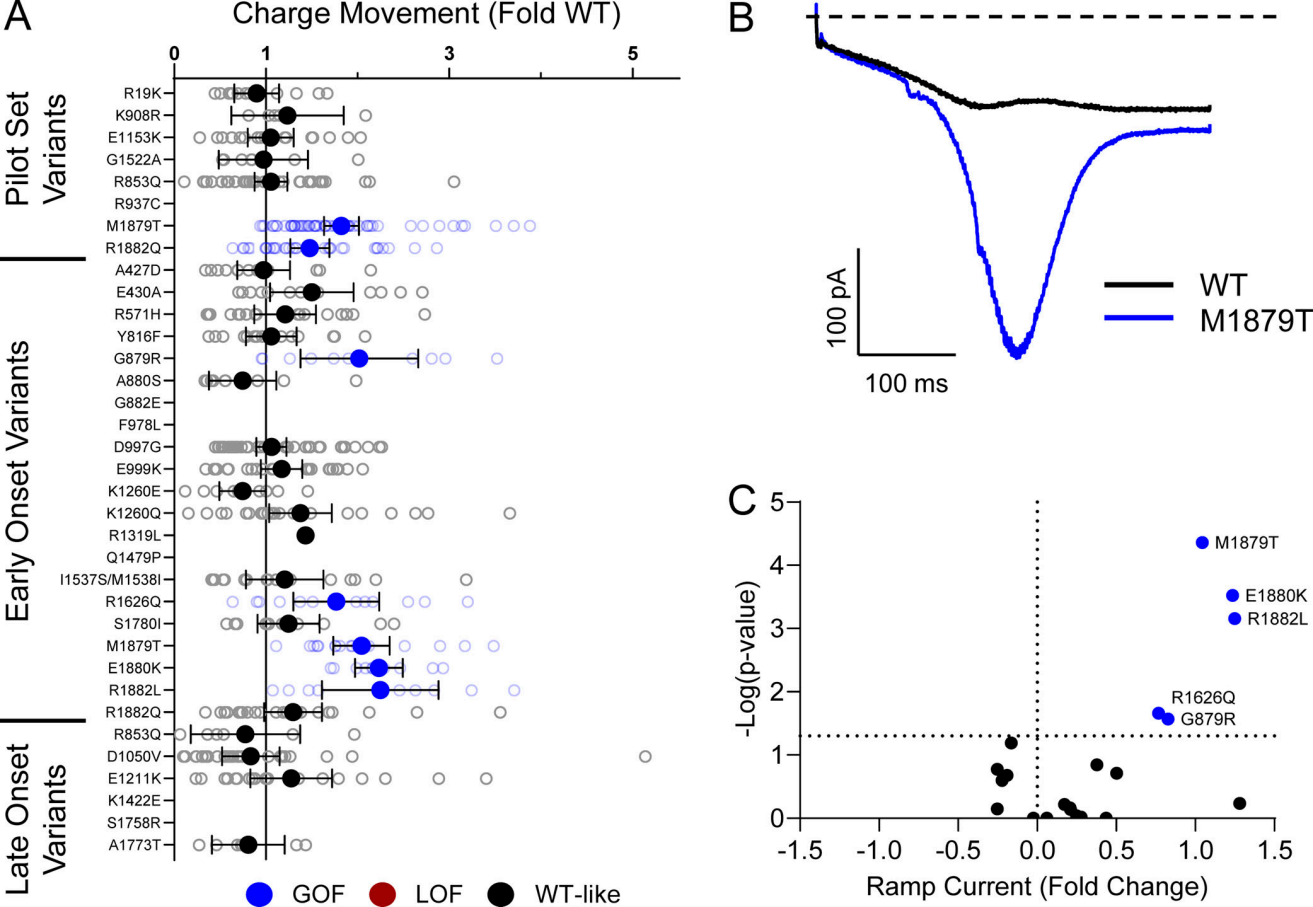
**Na**_**V**_**1.2 variants alter inactivation ramp currents. (A)** Average deviation of ramp currents from WT Na_V_1.2A for disease-associated variants. Data are plotted as mean ± 95% CI. **(B)** Average traces for WT Na_V_1.2A and a GOF variant, M1879T. **(C)** Volcano plot highlighting variants significantly different from WT. Red symbols denote LoF and blue symbols denote GoF variants classified based upon ramp currents significantly different from WT channels (*n* = 5–45). Data were collected from two to four separate 384-well automated patch clamp experiments, and statistical comparisons were performed using a Kruskal–Wallis test followed by Dunn’s post-hoc test for multiple comparisons. Exact P values are presented in Tables S3 and S4.

Domain IV in Na_V_ channels contributes to the voltage dependence of channel activity and to the time course for the onset of channel inactivation. Thus, in addition to shifts in voltage dependence of inactivation, variants in domain IV and the C-terminus may slow the onset of channel inactivation, which can lead to a slower time course of inactivation (reflected by larger time constants) and larger currents during a depolarizing voltage ramp protocol (Fig. S1). Indeed, 7 of the 11 variants with slower inactivation kinetics reside in these domains (I1537S/M1538I, R1626Q, A1773T, M1879T, E1880K, R1882L, and R1882Q; Fig. 5 and Table S4). Four of these variants (R1626Q, E1880K, M1879T, and R1882L) also showed larger ramp currents compared with WT (Fig. 6 and Table S4). One variant, G879R, exhibited a larger ramp current compared with WT, even though the onset of inactivation was slightly faster than WT. However, this larger ramp current was attributed to a larger persistent sodium current compared with WT (Fig. S6). We also measured frequency-dependent rundown (Fig. S6) and recovery from inactivation (Fig. S7) for each variant. While some variants showed GoF effects, LoF was the predominant phenotype for recovery from inactivation and frequency-dependent rundown.

**Figure 7.**
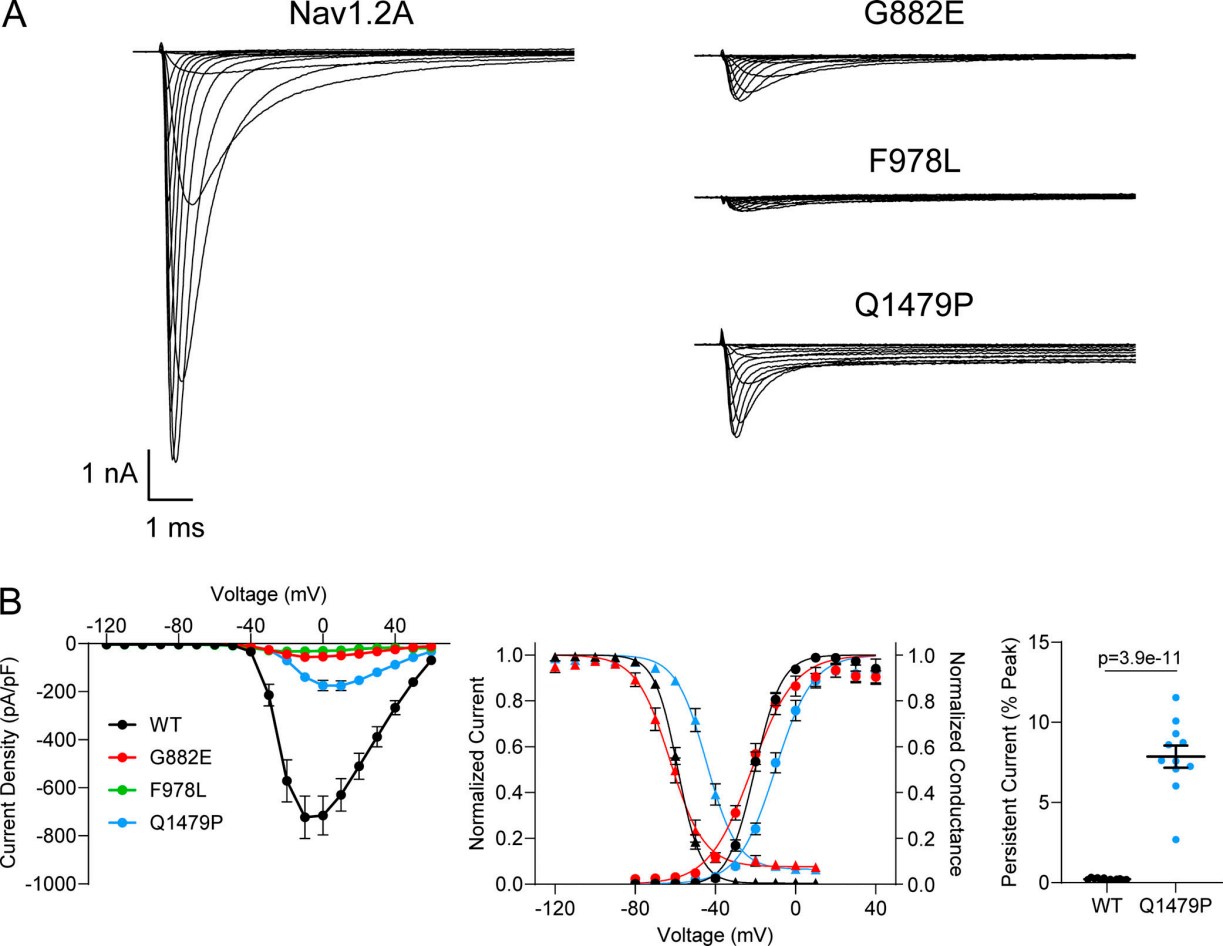
**Validation of difficult-to-record Na**_**V**_**1.2 variants using manual patch clamp. (A)** Average whole-cell current traces of WT and three difficult-to-characterize variants (G882E, F978L, and Q1479P) recorded using manual patch clamp. **(B)** Summary current–voltage relationship (left), voltage dependence of activation and inactivation (middle), and persistent current (right) of Na_V_1.2A variants recorded using manual patch clamp. All data were from 9 to 19 cells and are plotted as mean ± SEM. Data were collected from three individual transfections, and statistical analyses were performed using Student’s *t* test.

A small number of variants were challenging to evaluate using automated patch clamp due to either poor viability after electroporation or very small whole-cell currents that could not be distinguished from endogenous currents. We used manual patch-clamp recording to validate the phenotypes of three variants, G882E, F978L, and Q1479P. Both G882E and F978L exhibited very small currents compared with WT when recorded using either automated or manual patch clamp (Fig. 7, A and B). Although whole-cell current amplitude was small for G882E, they were large enough to reliably measure voltage dependence of activation and inactivation, and similar results were obtained from automated and manual patch-clamp recording. Voltage dependence of activation for G882E was similar to that of WT (WT: −21.5 ± 2.3 mV, *n* = 19; G882E: −21.1 ± 3.7 mV, *n* = 10; P = 0.9073), while exhibiting a hyperpolarized shift in voltage dependence of inactivation (WT: V_½_ = −58.4 ± 1.8 mV, *n* = 19; G882E: V_½_ = −63.3 ± 3.9 mV, *n* = 10; P = 0.0193; Fig. 7 B).

Cells electroporated with Q1479P had poor viability compared to cells electroplated with WT Na_V_1.2A. Automated patch-clamp recordings showed smaller currents than WT but indicated a large persistent sodium current that could potentially be toxic to cells. Manual patch-clamp recording revealed that while the peak whole-cell sodium currents were indeed smaller than that of WT, persistent current was 38 times larger than WT when measured as a percentage of peak current (WT: 0.2 ± 0.04%, *n* = 12; Q1479P: 7.7 ± 1.5%, *n* = 9; P < 0.00001; Fig. 7 B) and eightfold larger than WT when measured as current density (WT: −2.3 ± 1.3 pA/pF; Q1479P: −18.2 ± 9.5 pA/pF; P = 0.0048). Manual recording of Q1479P also revealed depolarized shifts in voltage dependence of activation (WT: V_½_ = −21.5 ± 2.3 mV *n* = 19; Q1479P: V_½_ = −10.2 ± 5.5 mV, *n* = 11; P < 0.0001) and voltage dependence of inactivation (WT: V_½_ = −58.4 ± 1.8 mV, *n* = 19; Q1479P: V_½_ = −44.2 ± 5.3 mV, *n* = 11; P < 0.0001, Fig. 7 B).

### Comparison of adult and neonatal splice isoforms

We studied variants associated with early-onset DEE in the neonatal Na_V_1.2N splice isoform (Figs. S8, S9, S10, S11, S12, S13, and S14; and Table S5) and found a mix of GoF and LoF properties, similar to that observed for the variants expressed in the canonical Na_V_1.2A isoform. There are a few variants for which splice isoform impacts the functional properties differently. For example, both R571H and K1260E expressed in the neonatal isoform exhibited hyperpolarized voltage dependence of activation compared to WT (Fig. S14), whereas expression in Na_V_1.2A was associated with WT-like activation voltage-dependence for R571H and K1260E. Additionally, while R853Q exhibited a greater sensitivity to frequency-dependent rundown in the adult isoform, its behavior was WT-like in the neonatal isoform (Fig. S14).

### Integrated summary of Na_V_1.2 variant dysfunction

**Figure 8.**
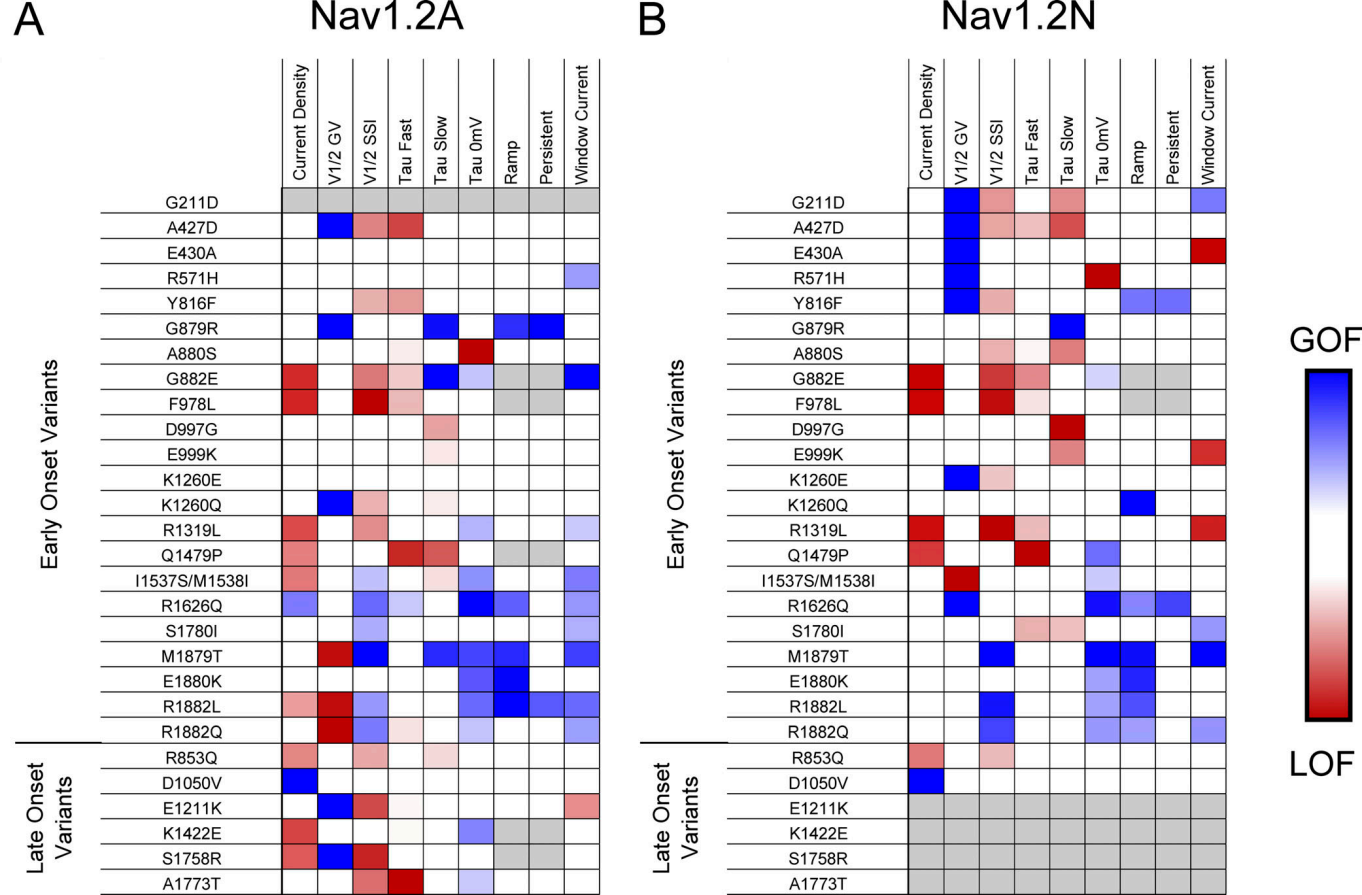
**Comparison of epilepsy-associated variants in the adult and neonatal isoforms of Na**_**V**_**1.2. (A and B)** Heat maps showing GoF (blue) and LoF (red) biophysical properties measured for epilepsy associated Na_V_1.2 variants in the (A) adult and (B) neonatal splice isoforms. Only properties that reached the threshold for statistical significance are highlighted.

Assigning the net effects of a variant as either GoF or LoF is challenging when considering the constellation of all measured functional properties. Complete LoF is easy to assign when there is no measurable sodium current (e.g., R937C). Similarly, variants with certain properties such as isolated enhanced persistent current, slower inactivation time course, or a large depolarizing shift in the voltage dependence of inactivation can be inferred to be GoF. However, most variants we studied exhibited mixed properties that are difficult to categorize according to this simple binary scheme. To provide a visual and integrated summary of functional properties for each variant ascertained in the two different splice variants, we constructed heat maps that scaled each biophysical parameter along a functional axis from loss to gain (Fig. 8). By displaying the data this way, many Na_V_1.2 variants appear to exhibit complex patterns of dysfunction with two or more properties exhibiting opposing effects, which is inconsistent with a simple binary overall functional effect.

## Discussion

The widespread use of genetic testing in medical practice and research has led to rapid growth in the number of genetic variants identified in ion channel genes associated with monogenic epilepsies. Determining the functional consequences of disease-associated ion channel variants has value in revealing pathogenic mechanisms, contributing to understanding genotype–phenotype relationships, and helping with the assessment of pathogenicity. However, the explosion in genetic data makes the functional evaluation of individual variants using traditional whole-cell patch clamp techniques insufficient, and techniques with more scalable throughput are required to meet demand.

In this study, we demonstrate successful use of automated patch-clamp recording to determine the functional properties of several *SCN2A* variants expressed in two splice isoforms. While traditional voltage-clamp techniques are considered to be the gold standard for evaluating ion channel function, automated patch clamp offers specific advantages that strengthen experimental rigor including higher throughput, the ability to directly compare WT and variants in parallel, and unbiased cell selection (Vanoye et al., 2021). Furthermore, the higher throughput achievable with automated patch clamp allows for greater numbers of variants to be studied under standardized experimental conditions, thus avoiding inter-laboratory heterogeneity. For this study, we investigated 28 distinct variants with most expressed in two splice isoforms (total 51 constructs) and report data recorded from nearly 6,000 individual cells. This work complements our prior efforts using automated patch clamp to determine the functional consequences and pharmacology of other disease-associated ion channels (Vanoye et al., 2018, 2022; Gertler et al., 2019; Kang et al., 2019).

We did observe some differences in Na_V_1.2 biophysical properties ascertained by the two methods, most notably smaller peak current density (Fig. S2 B) and slower inactivation kinetics (Fig. S2 F) with automated patch-clamp recording. We attributed smaller current density to a cell-selection bias inherent with manual patch-clamp recording, and the slower inactivation kinetics may be explained by a greater degree of series resistance compensation feasible with manual patch clamp due to lower access resistance. However, because all variants are compared with the WT channel assayed in parallel, intrinsic differences between automated and manual patch-clamp recording do not affect our conclusions.

Our evaluation of a cohort of disease-associated *SCN2A* variants revealed a spectrum of Na_V_1.2 dysfunction that was not easily parsed into the binary categories. When considering multiple biophysical parameters, many variants exhibit a constellation of dysfunctional properties that represent mixed GoF and LoF. Good examples of mixed patterns of dysfunction include R1882L and R1882Q. When expressed in Na_V_1.2A, both variants exhibit depolarizing shifts in activation voltage-dependence combined with slower kinetics of fast inactivation, which represent opposing effects on channel function. Perhaps given the complexity of *SCN2A*-related clinical phenotypes and that physiological function of *SCN2A* is developmentally regulated, complex patterns of variant channel dysfunction are not unexpected. Our finding of mixed functional properties suggests a need for a more nuanced classification of *SCN2A* variant dysfunction. Specifically, our results imply that the GoF versus LoF paradigm oversimplifies variant effect on channel function and does not fully capture the complete physiological impact of channel dysfunction. Our findings indicate that a more nuanced framework for describing and reporting the function effects of ion channel variants will be valuable.

Our data further emphasize the need to consider which parameters are the key drivers of channel dysfunction and abnormal neuronal physiology. Other scalable experimental approaches such as computational neuronal action potential modeling (Ben-Shalom et al., 2022) or dynamic action potential clamp recording (Berecki et al., 2018) may be valuable to determine the net effect of mixed dysfunctional properties on neuronal excitability. While these strategies are beyond the scope of our current study, the data we generated will be valuable to inform these complementary approaches.

Differences in *SCN2A* variant function may segregate with the age of onset of epilepsy or correlate with later onset neurodevelopmental disorders. Among the variants we studied that were associated with early onset seizure disorders, we observed functional properties consistent with an overall GoF (e.g., R1626Q, M1879T), mixed patterns of dysfunction, or overt LoF (e.g., F978L). Similarly, among variants associated with later onset seizures or a neurodevelopmental disorder without seizures (e.g., S1758R), we observed mixed functional properties, although channel LoF was the most prevalent effect (Fig. 8). The outlier was D1050V, which exhibited significantly larger peak current density than WT channels without differences in voltage-dependent or kinetic properties. This variant affects a residue in the D2–D3 cytoplasmic domain near an ankyrin binding site. A recent study provided evidence that loss of ankyrin-B scaffolding of Na_V_1.2 in cortical neurons phenocopies the neurophysiological defects observed in *Scn2a* haploinsufficient mice (Nelson et al., 2022 *Preprint*). These observations raise the possibility that D1050V disrupts Na_V_1.2 interactions with ankyrin and mimics a channel LoF. Future investigations of the cell surface expression or localization of *SCN2A* variants in neurons can test this hypothesis.

*SCN2A* undergoes developmentally regulated alternative mRNA splicing that generates transcripts containing one of two mutually exclusive alternative exons encoding a portion of the first domain voltage-sensor (S3 and S4 helices) with slight differences in amino acid sequence (Copley, 2004; Gazina et al., 2010). The neonatal-expressed Na_V_1.2N splice isoforms predominate during early development whereas transcripts containing the alternative (adult-expressed Na_V_1.2A) exon are expressed beginning in the postnatal period (Liang et al., 2021). We previously reported functional differences between neonatal and adult splice Na_V_1.2 isoforms and pointed out that some epilepsy-associated variants exhibit distinct patterns of dysfunction in the two isoforms (Thompson et al., 2020). Two C-terminal variants (R1882Q, R1882L), which are associated with neonatal onset epileptic encephalopathy, exhibit GoF profiles when expressed in the neonatal isoform but a more mixed profile in the adult isoform.

In summary, we conducted a systematic functional evaluation of a large cohort of disease-associated *SCN2A* variants using an optimized automated patch-clamp recording strategy. The scale of this work allowed us to compare the function of variants associated with different disease phenotypes and to examine how functional properties vary between developmentally regulated splice isoforms. Our findings illustrate a prevalence of complex biophysical effects for many variants suggesting that a simple binary scheme to classify variants as either GoF or LoF is insufficient to capture the full spectrum of variant channel dysfunction. The higher throughput capabilities of automated patch clamp enable greater standardization and more robust experimental rigor that is well suited to assessing *SCN2A* variant pathogenicity.

## Supplementary Material

Table S1 shows mutagenic SCN2A primers.

Table S2 shows comparison of Na_V_1.2A biophysical properties.

Table S3 shows biophysical properties of Na_V_1.2A population and epilepsy-associated variants.

Table S4 shows biophysical properties of Na_V_1.2A epilepsy-associated variants.

Table S5 shows biophysical properties of Na_V_1.2N epilepsy-associated variants.

## Data Availability

All analyzed data presented in this study are available in Tables S2, S3, S4, and S5.
