# Supplementary material for: Epilepsy-associated SCN2A (NaV1.2) variants exhibit diverse and complex functional properties
Source: J Gen Physiol. 2023 Aug 14;155(10):e202313375. doi: 10.1085/jgp.202313375 (PMC10424433; doi:10.1085/jgp.202313375)
Supplement: Table S1 — shows mutagenic SCN2A primers. [file JGP_202313375_TableS1.docx]

| **Variant** | **Set** | **Splice Isoform** | **Forward Primer** | **Reverse Primer** |
| --- | --- | --- | --- | --- |
| R19K | Training | Adult | TTTACCA**A**GGAATCCCTTGCTGCTATTGAACAACGC | AGGGATTCC**T**TGGTAAAGAAGCGGAAGCTGTCAGGTC |
| G211D | Test | Neonatal | TGTAAACCTAG**A**CAATGTTTCAGCTCTTCGAACTTTCAGAGTC | CATTG**T**CTAGGTTTACAAATTCTGTTACATACGCAAAAGTAATG |
| A427D | Test | Adult/Neonatal | CATGG**A**CTATGAGGAACAGAATCAGGCCACATTGG | GTTCCTCATAG**T**CCATGGCCACCACAGCCAAGATC |
| E430A | Test | Adult/Neonatal | TGAGG**C**ACAGAATCAGGCCACATTGGAAGAGGC | CCTGATTCTGT**G**CCTCATAGGCCATGGCCACCAC |
| R571H | Test | Adult/Neonatal | TCCAAGAC**A**CAACAGTAGGGCGAGCCTTTTCAGC | TACTGTTG**T**GTCTTGGAGAGAAAAGGGAGCCACG |
| Y816F | Test | Adult/Neonatal | CCAT**T**TTATTACTTTCAAGAAGGCTGGAATATTTTTGATGGTTTTATTG | TGAAAGTAATAA**A**ATGGATCCATGGCAATTATCTTGAGAAACATTTCTG |
| R853Q | Training/Test | Adult/Neonatal | ATCATTCC**A**GCTGCTCCGAGTTTTCAAGTTGGCAAAAT | GGAGCAGC**T**GGAATGATCGGAGAACTGACAATCCTTCC |
| G879R | Test | Adult/Neonatal | ATTGGCAATTCTGTG**A**GGGCTCTAGGAAACCTCACCTTGG | C**T**CACAGAATTGCCAATGATCTTAATTAGCATATTTAGAG |
| A880S | Test | Adult/Neonatal | TCTGTGGGG**T**CTCTAGGAAACCTCACCTTGGTATTGGC | CCTAGAG**A**CCCCACAGAATTGCCAATGATCTTAATTAGC |
| G882E | Test | Adult/Neonatal | CTCTAG**A**AAACCTCACCTTGGTATTGGCCATCATCG | GGTGAGGTTT**T**CTAGAGCCCCCACAGAATTGCCAATG |
| K908R | Training | Adult | TACA**G**AGAATGTGTCTGCAAGATTTCCAATGATTGTGAAC | CAGACACATTCT**C**TGTAGCTCTTACCAAAGAGCTGCATGCC |
| R937C | Training | Adult | GATCGTGTTC**T**GCGTGCTGTGTGGAGAGTGGATAG | GCACGC**A**GAACACGATCAGGAAGGAGTGGAAAAAGT |
| F978L | Test | Adult/Neonatal | TGAACCTC**C**TCTTGGCCTTGCTTTTGAGTTCCTTCAGT | GGCCAAGA**G**GAGGTTCAGAACCACTAGATTTCCAATCAC |
| D997G | Test | Adult/Neonatal | ATGATG**G**TAACGAAATGAATAATCTCCAGATTGCTGTGGG | CATTTCGTTA**C**CATCATCAGTGGCAGCAAGATTGTCAGAA |
| E999K | Test | Adult/Neonatal | TGATAAC**A**AAATGAATAATCTCCAGATTGCTGTGGGAAGG | TATTCATTT**T**GTTATCATCATCAGTGGCAGCAAGATTGTC |
| D1050V | Test | Adult/Neonatal | CTAAATAATAAAAAAG**T**CAGCTGTATTTCCAACCATACCACCATAG | **A**CTTTTTTATTATTTAGATCTTCAAGCGGTTTAATTTCATCTAAAGC |
| E1153K | Training | Adult | TCCCGCC**A**AGGGAGAACAGCCTGAGGTTGAACC | GTTCTCCCT**T**GGCGGGAGCTCCAATATCAACCG |
| E1211K | Test | Adult | ATTGGTTC**A**AAACCTTCATTGTCTTCATGATTCTGCTGAGC | GAAGGTTT**T**GAACCAATTGTGCTCCACTATCTTATAGCATG |
| K1260E | Test | Adult/Neonatal | ATGCTGCTA**G**AGTGGGTTGCATATGGTTTTCAAGTGTATTTTACC | ACCCACT**C**TAGCAGCATTTCCAGAATGAATATGTAAGTGAAAACC |
| K1260Q | Test | Adult/Neonatal | ATGCTGCTA**C**AGTGGGTTGCATATGGTTTTCAAGTGTATTTTACC | ACCCACT**G**TAGCAGCATTTCCAGAATGAATATGTAAGTGAAAACC |
| R1319L | Test | Adult/Neonatal | CCC**T**GTTTGAAGGAATGAGGGTTGTTGTAAATGCTC | CATTCCTTCAAAC**A**GGGACAAAGCTCTCAGTGGCCTC |
| K1422E | Test | Adult | GCCACGTTT**G**AGGGATGGATGGATATTATGTATGCAGCTG | CATCCCT**C**AAACGTGGCTACTTGAAGTAGAGACAGATATC |
| Q1479P | Test | Adult/Neonatal | CAACCAAC**C**GAAAAAGAAGTTTGGAGGTCAAGACATTTTTATGACA | TCTTTTTC**G**GTTGGTTGAAGTTATCTATGATGACACCAATGAAAAG |
| G1522A | Training | Adult | AG**C**AATGGTCTTTGATTTTGTAACCAAACAAGTCTTTGATATC | CAAAATCAAAGACCATT**G**CTTGGAATTTGTTCTGTGGAG |
| I1537S/M1538I | Test | Adult/Neonatal | ATATCAGCA**GCGC**CATGATCCTCATCTGCCTTAACATGGTCA | **GCGC**TGCTGATATCAAAGACTTGTTTGGTTACAAAATCAAAG |
| R1626Q | Test | Adult/Neonatal | ACCCTGTTCC**A**AGTGATCCGTCTTGCCAGGATTGGC | ATCACT**T**GGAACAGGGTAGGGGACACAAAATACTTT |
| S1758R | Test | Adult | TTTTTTGTC**C**GTTACATCATCATATCCTTCCTGGTTGTGG | ATGTAAC**G**GACAAAAAAGAAAATCCCAACAGATGGGTTCC |
| A1773T | Test | Adult | ATGTACATC**A**CGGTCATCCTGGAGAACTTCAGTGTTG | ATGACCG**T**GATGTACATGTTCACCACAACCAGGAAGG |
| S1780I | Test | Adult/Neonatal | ACTTCA**T**TGTTGCTACTGAAGAAAGTGCAGAGCCTC | AGTAGCAACA**A**TGAAGTTCTCCAGGATGACCGCGATG |
| M1879T | Training/Test | Adult/Neonatal | AATACAGA**C**GGAAGAGCGATTCATGGCATCAAACCC | GCTCTTCC**G**TCTGTATTCGAAGGGCATCCATCTCTCC |
| E1880K | Test | Adult/Neonatal | TACAGATG**A**AAGAGCGATTCATGGCATCAAACCCCTC | TCGCTCTT**T**CATCTGTATTCGAAGGGCATCCATCTCTC |
| R1882L | Test | Adult/Neonatal | GAAGAGC**T**ATTCATGGCATCAAACCCCTCCAAAGTCT | GCCATGAAT**A**GCTCTTCCATCTGTATTCGAAGGGCATC |
| R1882Q | Training/Test | Adult/Neonatal | GAAGAGC**A**ATTCATGGCATCAAACCCCTCCAAAGTCT | GCCATGAAT**T**GCTCTTCCATCTGTATTCGAAGGGCATC |

**Table S1.** Mutagenic SCN2A primers
